# Interfacial charge transfer on hierarchical synergistic shell wall of MXene/MoS_2_ on CdS nanospheres: heterostructure integrity for visible light responsive photocatalytic H_2_ evolution

**DOI:** 10.1186/s40580-024-00454-1

**Published:** 2024-12-02

**Authors:** Kugalur Shanmugam Ranjith, Ali Mohammadi, Ganji Seeta Rama Raju, Yun Suk Huh, Young-Kyu Han

**Affiliations:** 1 Department of Energy and Material Engineering, Dongguk University-Seoul, Seoul, 04620 South Korea; 2 Department of Biological Sciences and Bioengineering, Nano Bio High-Tech Materials Research Center, Inha University, Incheon, 22212 South Korea

**Keywords:** Small layered MXene, Ternary heterostructure, Photocatalytic H_2_ evolution, CdS spheres, Photocorrosion

## Abstract

Energy scarcity and environmental pollution have prompted research in hydrogen generation from solar to develop clean energy through highly efficient, effective, and long-lasting photocatalytic systems. Designing a catalyst with robust stability and an effective carrier separation rate was achieved through heterostructure assembly, but certain functionalities must be explored. In this paper we designed a ternary heterostructure assembly of CdS nanospheres wrapped with hierarchical shell walls of layered MXene-tagged MoS_2_ nanoflakes, forming intimate interfaces through an in-situ growth process. An in-layered shell wall of MXene with surface-wrapped MoS_2_ nanoflakes as a core–shell assembly improved the photo-corrosion resistance and accelerated the production of photocatalytic H_2_ (38.5 mmol g^−1^ h^−1^), which is 10.7, 3.1, and 1.9 times faster than that of CdS, CdS–MXe, and CdS–MoS_2_ nanostructures, respectively. The apparent quantum efficiency of the CdS–MXe_2.4_/MoS_2_ heterostructure was calculated to be 34.6% at λ = 420 nm. X-ray and ultraviolet photoelectron spectroscopies validated the electronic states, energy band alignment, and work function of the heterostructures, whilst time-resolved photoluminescence measured the carrier lifespan to evaluate the effective charge migration in the CdS-MXe/MoS_2_ heterostructure. The dual surface wrapping of MXe/MoS_2_ over CdS nanospheres confirmed the structural durability that remained intact throughout the photocatalytic reaction, promoting approximately 93.1% of its catalytic property even after five repeatable cycles. This study examined how the MXene heterostructure template improves the catalytic efficiency and opens a new way to design MXene-based durable heterostructure catalysts for solar-energy conversion.

**Graphical Abstract:**

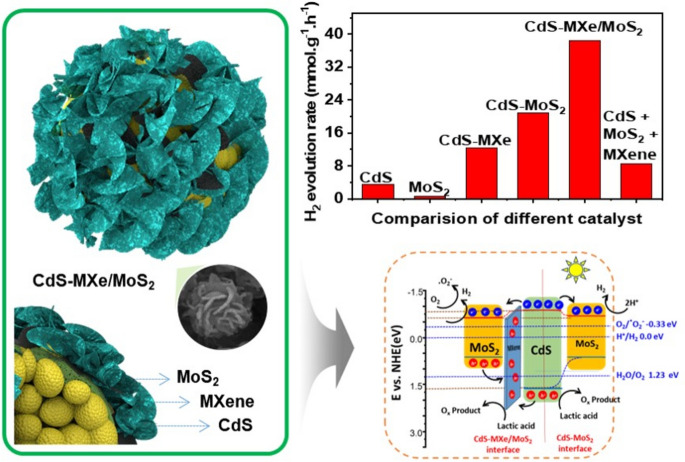

**Supplementary Information:**

The online version contains supplementary material available at 10.1186/s40580-024-00454-1.

## Introduction

Energy shortages and environmental pollution from fossil fuel consumption have prompted research into solar energy as a potential replacement because of its limitless, clean, and renewable nature [1, 2]. Following the discovery of electrodes for water photoelectrolysis, semiconducting photocatalysts have attracted significant attention for their ability to convert solar energy into the necessary chemical energy [3, 4]. Solar-induced H_2_ evolution has attracted attention because of its recognition as a clean and green energy source, offering promising alternatives to fossil fuels [5, 6]. Nevertheless, developing a low-cost, efficient, and stable photocatalyst is critical because it requires excellent light adsorption capability, efficient carrier separation, and migration capability with a suitable band structure for reducing H_2_O to H_2_ [7]. In recent years, several studies have examined photocatalytic H_2_ evolution over a wide range of photocatalysts, including metal oxides [8, 9], metal sulfides [10, 11], organic frameworks [12, 13], and graphitic nitrates [14, 15]. On the other hand, heterostructures play a crucial role in overcoming the poor adsorption rate, carrier recombination, and low catalytic efficiency of the single-catalytic systems of many semiconductors [16]. Improving the efficiency of the photocatalytic process and the photogenerated carrier separation efficiency is the primary focus of modern photocatalytic H_2_ research [17].

Among the various photocatalytic semiconductors, CdS stands out because of its band gap (2.4 eV), which holds great promise for the evolution of photocatalytic H_2_ owing to its strong visible adsorption and suitable conduction band position (− 0.58 eV) [18]. On the other hand, the sluggish reaction kinetics of CdS, caused by agglomeration, depletion of reaction active sites, and carrier recombination, does not meet the requirements for an effective photocatalyst [19]. Furthermore, photocorrosion and reusable stability pose significant challenges to the bare CdS, resulting in a poor H_2_ production rate with low catalytic stability [20]. The pitfall of the catalytic system was overcome by introducing co-catalytic functionalities to optimize the carrier pathway and effective photogenerated carrier separation efficiency and maximize photocatalytic H_2_ evolution [21]. Decorating the co-catalytic functionalities on CdS has accelerated the photocatalytic reaction with a suitable co-catalyst that can reduce the hydrogen precipitation barrier and separate the photogenerated electrons, minimizing carrier recombination [22, 23]. The photocorrosion stability on CdS was improved by the heterostructure features that minimized surface oxidization, which promoted the structural stability of the catalyst [22]. Tagging a two-dimensional (2D) semiconducting interface and producing the unique nanolayer structure with a regulated layer distribution promoted light adsorption and carrier separation efficiency in photocatalytic H_2_ evolution [24, 25]. Although transition metal dichalcogenides-based heterostructures exhibited enhanced photocatalytic activity, the rapid recombination of electrons and holes limited the catalytic activity [26]. These significant challenges have been overcome by tagging the lamellar structured MoS_2_ over CdS to minimize the reduction potential and maximize the movement of negative charge carriers between the heterostructure interface [27]. The photogenerated electrons were trapped through the heterostructure interface and the sulfur edges, promoting the reactive active sites for proton reduction by tagging the stacked network of MoS_2_ on the CdS through the van der Walls interaction [28]. Wrapping the MoS_2_ nanoflakes in a hierarchical assembly on the CdS surface as a core–shell platform is an innovative process that enables the MoS_2_ to form an intimate contact, promoting interfacial charge carriers and shortening the photogenerated electron pathway towards the surface.

As an alternative co-catalyst, layered titanium carbide (Ti_3_C_2_) MXene has attracted considerable interest in its physicochemical properties with its unique morphology, surface conductivity, and multiple surface-active sites [29]. Following its discovery, the unique electrical and optical properties of MXene facilitated its widespread use in various fields such as photocatalysis, supercapacitors, electrocatalysis, batteries, and sensors [30, 31]. Fabricating layered MXene through an acid etching process would foster the formation of Ti vacancy states, promoting the electron-rich network [32]. Furthermore, as a hole migrator, MXene may promote hole mobility in the heterostructure assembly [33]. The heterostructure interface of MXene with the narrow band gap semiconductors promotes H_2_ evolution activity through the mechanistic electron transfer channel, light adsorption, and improvised active sites [34]. Tagging MXene with CdS as a heterostructure assembly significantly enhanced photocatalytic H_2_ evolution and NH_3_ synthesis, which was attributed to the excellent carrier migration with a suitable Fermi position [35]. Coupling MXene and MoS_2_ on CdS as a ternary network effectively promotes the photoresponsive activity and stability of the CdS nanostructures [36, 37]. To the best of the authors’ knowledge, specific ternary assemblies as a core–shell heterostructure with an in-layered MXene network in a comfortable fashion for photocatalytic H_2_ evolution have not been discussed.

In this study, ternary heterojunctions of MXene/MoS_2_ shell walls over CdS nanospheres as core–shell hierarchical spheres were constructed using a solvothermal process. Over the monodispersed CdS nanospheres, ultra-thin layered MXene was tagged through the electrostatic interaction, followed by solvothermal-grown MoS_2_ nanoflakes wrapped over the spherical surface. The layered feature of MXene wrapped over CdS nanospheres can capture photogenerated holes and improve the charge separation efficiency. Decorated MoS_2_ nanoflakes on the surface have a co-catalytic effect that brings electrons to the surface, making it an active site for photocatalytic hydrogen evolution. The dual shell wall strategy effectively separates the photogenerated electrons and holes with improvised active sites and light trapping ability, significantly enhancing the photocatalytic activity. Under visible irradiation, the photocatalytic hydrogen production rate of the ternary CdS–MXe_2.4_/MoS_2_ (38.5 mmol g^−1^ h^−1^) heterostructure was 10.7, 3.1, and 1.9 times faster than that of CdS, CdS–MXe, and CdS–MoS_2_ spheres, with an apparent quantum efficiency (AQE) of 34.6% at 420 nm. The carrier separation mechanism and photocatalytic H_2_ production were examined through various detailed physicochemical properties, and the mechanisms underlying the improvised catalytic performances were explored. This study examined a method to fabricate MXene-based ternary heterostructure composites as a solar energy harvester and potentially help design a very efficient and cost-effective photocatalytic system for H_2_ evolution.

## Experimental

### Preparation of ultra-thin MXene nanosheets

Layered MXene nanosheets were prepared using the etching process with LiF–HCl reported elsewhere [30]. Briefly, 20 mL of a 9 M hydrochloric acid solution was mixed with 2 g of lithium fluoride (LiF). The mixture was agitated for 15 min at room temperature. Subsequently, 2 g of Ti_3_AlC_2_ MAX powder (Carbon, Ukraine) was dispersed gradually in the above mixture and stirred at 40 °C for 72 h to etch the Al from the Ti_3_AlC_2_ selectively. Finally, the slurry was washed and centrifuged at 6000 rpm with water until the supernatant reached a pH ≈ 6. The supernatant was collected, placed into a freeze-dryer, and stored for later use. The ultrathin layered MXene nanosheets were prepared by mixing a certain amount of layered MXene with DMSO, and the homogeneous suspension was aged for 24 h. The suspension was centrifuged, cleaned with deionized (DI) water, and sonicated in a probe sonicator for 4 h in an ice bath under Ar purging to induce the delamination process. Finally, the ultra-thin MXene nanosheets were obtained by collecting the supernatant after centrifuging the suspension at 3500 rpm for 15 min, freeze-dried, and stored in a vacuum for further use.

### Synthesis of CdS nanospheres

The CdS spheres were synthesized using the reflex-based solution growth process. In detail, 1.2 g cadmium acetate dihydrate (Cd(CH_3_COO)_2_•2H_2_O) was added with 1 g of polyvinylpyrrolidone (PVP) in 80 ml of ethylene glycol (EG) and stirred at room temperature for 20 min. Subsequently, 1.8 g of thiourea (CH_4_N_2_S) was added, and the mixture was heated at 180 °C in a reflux condenser and maintained for 6 h before naturally cooling to room temperature. The precipitate was collected, centrifuged three to five times with ethanol and DI water, and dried in a vacuum at 60 °C for 2 h. The sample was annealed at 300 °C for 2 h in an Ar atmosphere with a heating ramp of 4 °C min^−1^ in a tubular furnace to improve the crystallinity of the CdS nanostructures.

### Synthesis of binary CdS–MXe nanospheres

The ultra-thin layered MXene was tagged on the CdS surface by surface functionalizing the CdS spheres with poly (diallyl dimethyl ammonium chloride) (PDDA), and a certain amount of MXene was attached electrostatically to the CdS surface. In detail, as-prepared CdS nanospheres (500 mg) were dispersed homogeneously in 20 ml of isopropanol. The dispersion was added with PDDA (15 μL) and placed in a shaker at 200 rpm for 2 h to produce positive surface functionality on the CdS surface. The dispersion was centrifuged and washed with isopropanol to remove the unreached and excess PDDA from the solution. The precipitate was redispersed in ethanol with a certain amount of MXene added dropwise into the CdS dispersion, shaken continuously at 120 rpm for 6 h, and dried under vacuum to prepare the CdS-tagged MXene-based heterostructure. The positive functionality of the CdS/PDDA favored the attachments of layered MXene nanosheets through electrostatic interactions, which leads to fabricated CdS–MXe heterostructures. The prepared samples were called CdS–MXe_x_ (x = 0.6, 1.2, 2.4, 3.6, 4.8, and 9.6 wt%), where x refers to the mass loading wt% of ultra-thin layered MXene to CdS spheres.

### Synthesis of ternary CdS–MXe/MoS_2_ nanospheres

MXene-interlayered CdS–MoS_2_ hierarchical nanospheres were prepared using a solvothermal process. Typically, 100 mg of MXene_2_-tagged CdS nanospheres were dispersed homogeneously in 60 ml of EG/DI water (1:1 ratio) by stirring for 5 min. The sodium molybdate (Na_2_MoO_4_·2H_2_O, 0.4 g) and CH_4_N_2_S (0.8 g) were mixed with the above dispersion as precursors for MoS_2_ growth. The well-dispersed solvent was heated to 200 °C for 10 h in a Teflon-lined autoclave (100 mL). After cooling, the precipitate was collected, washed with ethanol and DI water, and dried at 60 °C for 12 h in a vacuum. The collected sample was annealed at 300 °C for 2 h in an Ar atmosphere with a heating ramp of 4 °C min^−1^ in a tubular furnace to improve the crystallinity of the ternary heterostructure nanostructures. For comparison, the binary sample without MXene (CdS–MoS_2_) and bare MoS_2_ were prepared under similar operation conditions, respectively. CdS–MXe_x_ (x = 2.4) was used as an optimal heterostructure for MoS_2_ growth because of the high light adsorption and catalytic activity. While calculating the mass loading, approximately 34–37% of MoS_2_ was loaded over the CdS surface, as quantified through the controlled experimental process.

### Characterization

The crystal phase of the ternary composites was examined by powder X-ray diffraction (XRD, PANalytical X’Pert Pro multipurpose X-ray diffractometer) using Cu Kα irradiation (λ = 0.15406 nm). Raman spectroscopy was conducted using a 532-nm laser Raman microscope (FEX, NOST), and X-ray photoelectron spectroscopy (XPS, Thermo Scientific spectrometer) was tested using Al Kα radiation (1486.6 eV) to quantify the electronic states and composition of the heterostructures. The morphology of the ternary heterostructures was quantified by high-resolution scanning electron microscopy (HRSEM, SU 8010; Hitachi) and high-resolution transmission electron microscopy (FETEM, JEM-2100F; JEOL), and the elemental composition and maps were obtained by energy dispersive spectroscopy (EDS, linked to FETEM). Fourier transform infrared (FTIR, JASCO FTIR 6600) spectroscopy was used to detect surface functionalities at 400 to 4000 cm^−1^. The optical absorption properties were quantified by ultraviolet–visible (UV–Vis) diffused reflectance spectroscopy (DRS, JASCO V-770), and the photoluminescence (PL) was analyzed using a fluorescence spectrophotometer (FEX, NOST) under the excitation of 375 nm. The time-resolved photoluminescence (TRPL) was analyzed using a Horiba Hobin Yuon fluorescence spectrophotometer (iHR320) at an emission wavelength of 405 nm. The specific surface area and the pore size distribution were quantified from the N_2_ adsorption–desorption isotherm at 77 K on a Tristar ASAP 2020. Inductively coupled plasma–optical emission spectroscopy (ICP–OES; Optima 7300 DV, PerkinElmer, USA) was used to quantify the Ti_3_C_2_ loading in the heterostructure samples. UV photoelectron spectroscopy (UPS, ThermoFischer EscaLab 250 Xi spectrometer) was used to determine the work function and Fermi level using Helium Iα as the excitation source.

### Photocatalytic H_2_ evolution

Photocatalytic H_2_ production was quantified using a 150 mL Pyrex glass reactor with a top quartz window and an external water-cooling system. The Xenon lamp (100 W with the 420 nm cut-off filter) was placed above the reactor as a light source, and the temperature of the photocatalytic reactor was maintained at 5 °C throughout the experiment. The illumination area was approximately 28.3 cm^2^, and the light source was placed above 12 cm from the top of the reactor solution. In detail, the 20 mg of photocatalyst was dispersed with 80 mL of an aqueous lactic acid (LA) solution (10 v%). After the dispersion, the reactor was sealed, and the chamber was evacuated, followed by degassing with high-purity Ar to complete air removal. The catalyst was dispersed with the reaction solution and magnetically stirred in the reaction cell to avoid photocatalyst sedimentation. At 30-min intervals, 0.1 mL of the resulting gas was extracted, and the H_2_ production rate was quantified on gas chromatography (GC, Agilent 6890N) equipped with the 5 Å molecular sieve column with a thermal conductivity detector (TCD). The apparent quantum yield (AQY) of photocatalytic H_2_ production relative to different monochromatic light filters (420, 460, 510, 560, and 620 nm) was calculated [38].

### Photoelectrochemical measurements

The photoresponsive electrochemical properties were examined using an electrochemical workstation (Metrohm, Auto Lab: PGSTAT302N) through the transition photocurrent, electrochemical impedance, and linear sweep voltammetry curves with the standard three-electrode measurements. The CdS-based samples were coated on an FTO substrate and used as a working electrode. The working electrode was prepared on the FTO substrate (1 cm × 1 cm), as reported in the previous report [39]. Pt foil and the saturated calomel electrode (SCE) were used as the counter and reference electrodes, respectively. The transition photocurrent measurements were taken with 0.5 M Na_2_SO_4_ as an electrolyte, and the 100 W Xe light source (PhotoFluor II) with the 420 nm cut-off filter was used as a light source for the photoirradiation. EIS analysis was performed at the open circuit potential with a frequency range of 100 kHz to 0.01 Hz using a mixture of 0.025 M potassium ferricyanide as an electrolyte.

## Result and discussion

**Scheme 1:**
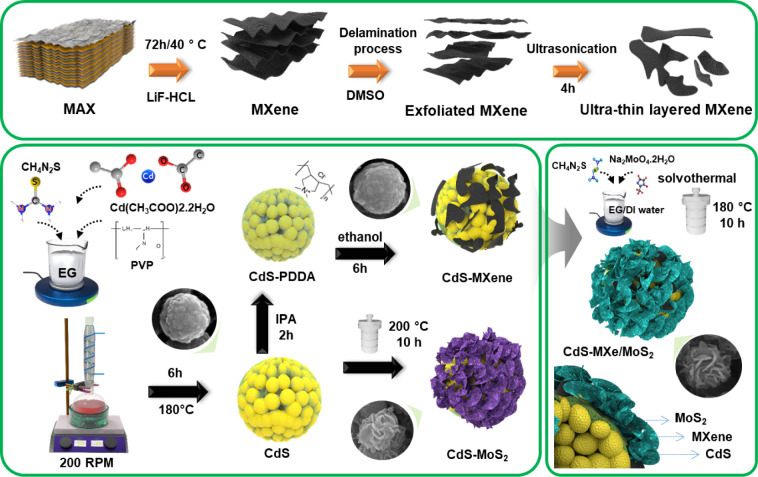
Schematic illustration for the fabrication of ternary CdS–MXe/MoS_2_ hierarchical nanospheres

**Fig. 1:**
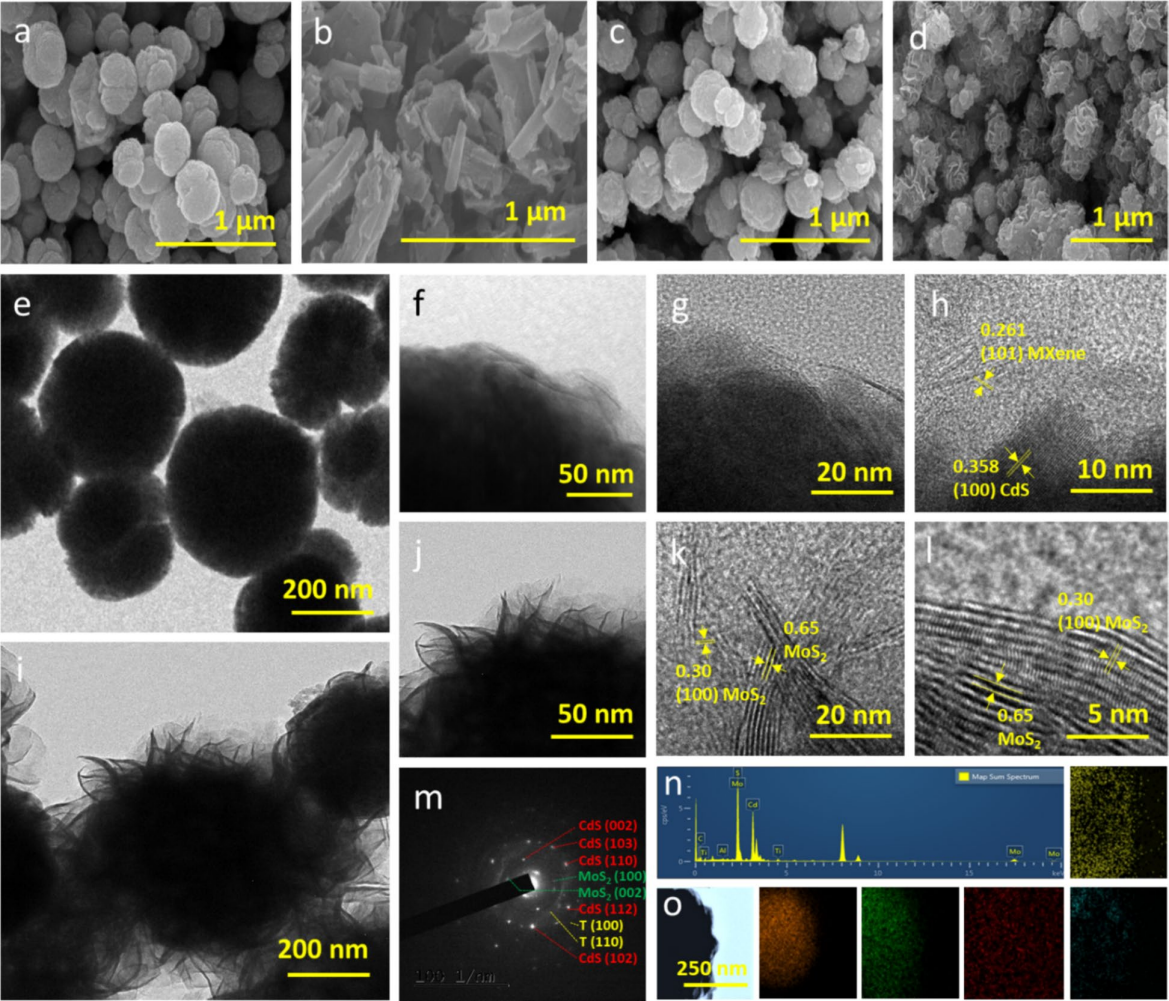
SEM images of **a** CdS, **b** MXene, **c** CdS–MXe, and **d** CdS–MXe/MoS_2_. The FETEM and HRTEM images of **e**–**h** CdS–MXe and **i**–**l** CdS–MXe/MoS_2_. **m** SAED pattern of CdS–MXe/MoS_2_ **n**, **o** EDAX spectra and HAADF-STEM mapping images of the CdS–MXe/MoS_2_ with the distribution of its Cd, S, Ti, C, and Mo atoms

Scheme 1 presents a schematic flow diagram for synthesizing the ternary CdS–MXe/MoS_2_ heterostructure nanospheres. The solution-grown CdS nanospheres were tagged with delaminated ultra-thin layered MXene through surface functionalization. Subsequently, MoS_2_ nanoflakes were grown over the CdS–MXe heterostructure using a solvothermal process, establishing intimate contact with the CdS–MXe/MoS_2_-based ternary heterostructure. The morphology and microstructural composition of the CdS, CdS–MXe, and CdS–MXe/MoS_2_ nanostructures were analyzed by HRSEM and FETEM. Figure 1a presents the as-prepared CdS nanospheres, with a particle diameter of approximately 200 nm and exhibit an agglomerated structural assembly. The MXene was fabricated by the selective etching of Al on the MAX phase, resulting in a layered structure (Fig. S1), which proceeded with an exfoliation process to obtain the ultra-thin MXene nanosheets. With the size between 150–500 nm (Fig. 1b), the delaminated layered MXene sheets had a flat, smooth surface, which was 10 times smaller than the as-prepared MXene sheets (Fig. S1). Figure 1c shows the surface tagging of ultra-thin layered MXene on the CdS spheres, using the functionality of PDDA to maximize the intimate contact of MXene on the CdS spheres.

The ternary composite produces an aggregated spherical morphology with a hierarchical surface texture, leading to MoS_2_ growth on the CdS–MXe spheres (Fig. 1d). MoS_2_ grew over the CdS spherical surface, with its surface facets measuring around 150 nm. No significant morphological differences were observed between the CdS/MoS_2_ and CdS–MXe/MoS_2_ heterostructures (Fig. S2), apart from the slight surface density of the MoS_2_ on MXene functionalized heterostructures. Compared to the pristine CdS, the composite feature experienced less agglomeration because of the lamination effect of MXene and MoS_2_ on its spherical surface. TEM of CdS showed that the nanograins were combined to form a spherical structure with an aggregated grain size distribution of approximately 10 nm (Fig. S3). While tagging the MXene with CdS, the 2D ultra-thin layered MXene was surface-wrapped over the CdS sphere, which would form a thin shell wall on the CdS through surface functionalization (Fig. 1e–h). The wrapping of ultra-thin layered features on the surface of CdS spheres resulted in the intimidating heterostructure interface of CdS–MXe nanospheres. The mapping results and high-angular dark field TEM (HAADF TEM) analysis showed that MXene was surface-wrapped over the CdS spheres, exhibiting an elemental distribution of Cd, S, and surface-functionalized Ti and C (Fig. S4). The HRTEM image of CdS–MXe (Fig. 1h) showed the lattice spacing of 0.358 nm and 0.261 nm, which was attributed to the CdS(100) and MXene(101) crystal faces, respectively. Figure 1i–k shows the hierarchical assembly of the ternary phase CdS–MXe/MoS_2_ heterostructure assembly, where the MoS_2_ was intimately grown over the sphere assembly. The hierarchical array growth of MoS_2_ over MXe/CdS resulted in the core–shell assembly of CdS–MoS_2_ with the MXene-based interlayer acting as a bridging network. The layered surface (Fig. 1l) had a lattice spacing of 0.65 nm and 0.30 nm, representing the layer spacing and (100) crystal planes of MoS_2_ nanoflakes. Without the MXene-based interlayer, the CdS–MoS_2_ core–shell nanospheres exhibited similar morphology with a slight density variation on the hierarchical shell wall (Fig. S5). The heterostructure spherical assembly showed the integrity of CdS with MXene and MoS_2_, forming a ternary assembly similar to the core–shell network. Selected area electron diffraction showed the crystal plains of CdS(100), MoS_2_(002), and MXene, respectively (Fig. 1m), indicating the successful fabrication of ternary assemblies. The EDAX spectra (Fig. 1n) and image mapping analysis (Fig. 1o) revealed the in-layered loading of MXene on the CdS surface with the surface wrapping of MoS_2_ and the elemental distribution of Cd, S, C, Ti, and Mo on the heterostructure surface.

**Fig. 2:**
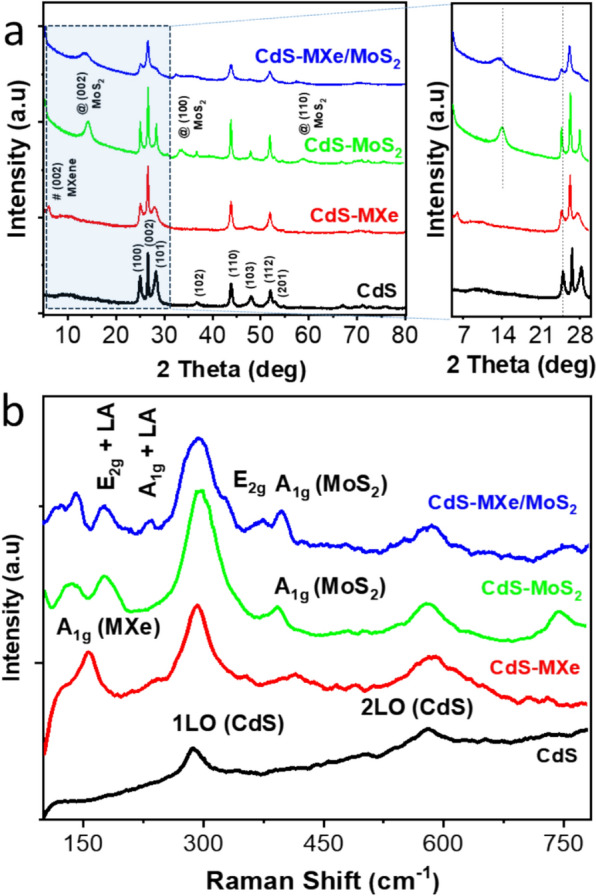
**a** XRD patterns and **b** Raman analysis of the CdS, MXene, CdS–MXe, CdS/MoS_2_, and CdS–MXe/MoS_2_ samples

**Fig. 3:**
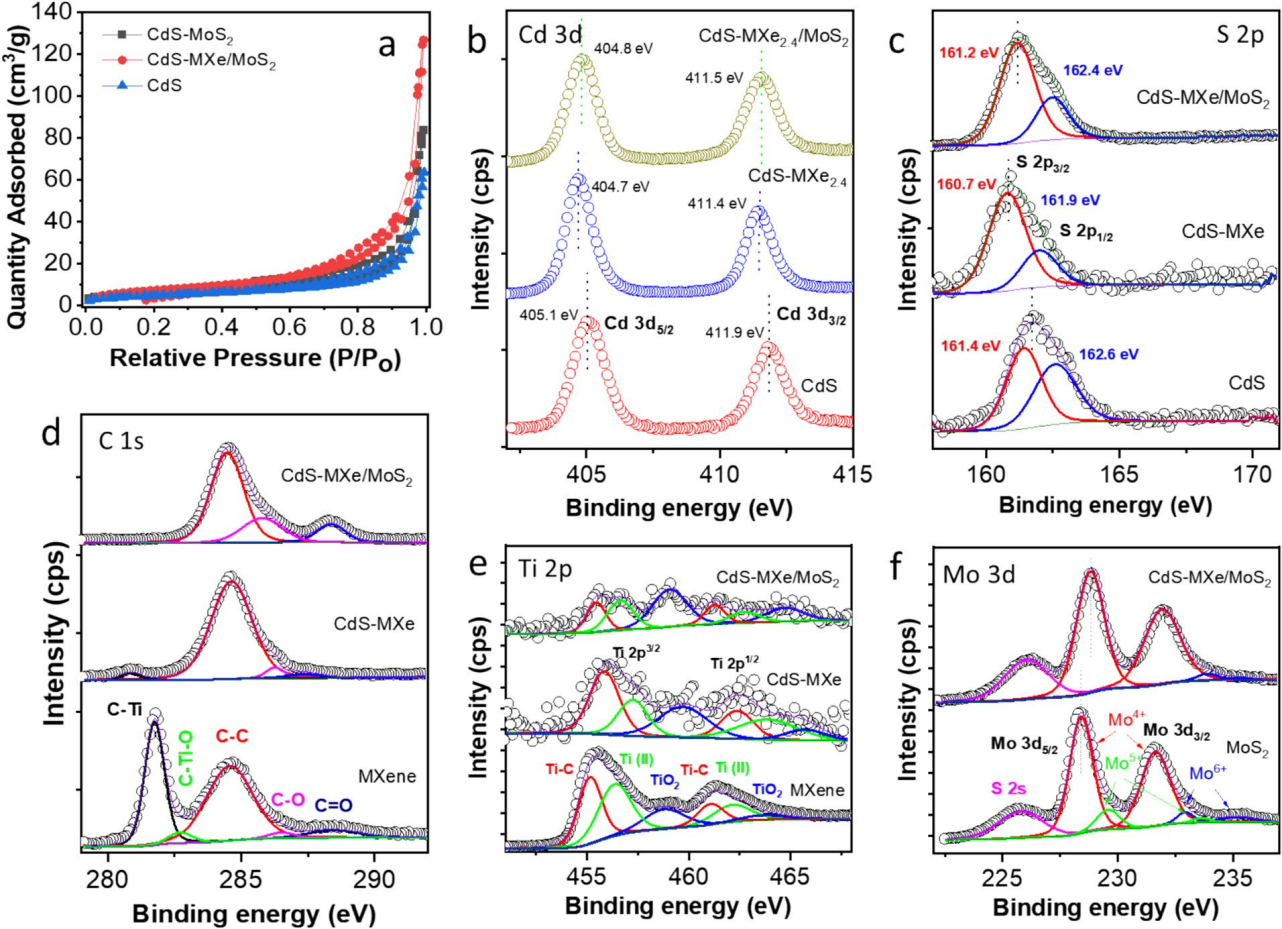
**a** N_2_ adsorption–desorption isotherm of CdS, CdS–MoS_2_, and CdS–MXe/MoS_2_ samples. High-resolution XPS spectra of **b** Cd 3d and **c** S 2p of CdS, CdS–MXe, and CdS–MXe/MoS_2_. **d** C 1 s and **e** Ti 2p of MXene, CdS–MXe, CdS–MXe/MoS_2_, and **f** Mo 3d of MoS_2_ and CdS–MXe/MoS_2_ samples

The structural phase of the samples was analyzed using XRD, Raman, and XPS analysis. The XRD pattern of the pristine MAX revealed an XRD peak at 9.6°, which was indexed to the (002) lattice plane, as shown in Fig. S6. The peak shifted further to 7.7° due to the selective etching process, indicating an extended, large interlayered spacing during the fabrication process of MXene. With the function of the delamination process, the peak shifted further to 5.7°, showing an extended, large interlayered spacing [40]. The as prepared, CdS nanospheres show the hexagonal structural phase (JCPDS card: 41–1049) (Fig. 2a), with the XRD peaks at 24.9, 26.4, 27.9, 36.4, and 43.8° being consistent with the (100), (002), (101), (102), and (110) planes, respectively. Creating the PDDA functionality resulted in the change in zeta potential from – 16.2 to 3.5 mV for CdS under neutral conditions (Fig. S7a), and the FTIR data (Fig. S7b) indicated the presence of C-H related functionalities on the CdS surface, which confirms the functionalization of PDDA. The XRD results (Fig. S7c) confirm that the PDDA functionality of CdS nanostructures influences the loading density of MXene compared to non-functionalized CdS nanospheres, leading to a favorable interaction between MXene and CdS through the PDDA functionalization. The function of tagging the MXene caused the CdS-related peaks to shift towards lower angles, interacting with the CdS surface. The 2θ value, which represents the (002) plane of Ti_3_C_2_ in CdS-MXe shifted from 5.8° to 7.1° as the MXene concentration increased (Fig. S8a), signifying a reduction in interplanar spacing due to the extensive wrapping of MXene on the CdS surface. The FTIR spectra of the different wt% of MXene-loaded CdS were characteristic of the composite feature’s surface functionality (Fig. S8b). The surface functionality of the CdS–MXe composites was consistent with the CdS-related peaks. The MXene-related peaks did not appear strong in the samples at lower concentrations because of the high dispersion and thin-layered features. Meanwhile, as the wt% was increased above 2.4 wt%, peaks appeared at 2920 cm^−1^ and 1105 cm^−1^, which were related to the C–H vibrations and C–F stretching vibrations of MXene nanosheets. After the solvothermal process, MoS_2_-related XRD peaks appeared on the composite samples (Fig. 2a), which exhibited new peaks at 14.3°, 33.3°, and 58.9° belonging to the (002), (101), and (110) lattice planes of the 2H-MoS_2_ hierarchical layered crystal structure (JCPDS No. 37–1492), respectively. Compared to the pristine samples, the composites experienced a shift in the XRD peaks, which led to the interaction of the hetero interface on the composite surface. The existence of MXene and MoS_2_ on the CdS surface was identified by Raman analysis (Fig. 2b). The two characteristic peaks at 286.8 and 583.8 cm^−1^ were assigned to the longitudinal optical (LO) modes of hexagonal CdS [41]. The CdS–MXe heterostructures showed typical peaks at 205 cm^−1^, corresponding to the out-plane vibrations of Ti and C atoms. The intense peak at 157 cm^−1^ related to the oxidized Ti_3_C_2_ could be due to the increased laser power on the layered surface [42]. The D and G bands were observed at 1352 and 1567 cm^−1^, respectively, representing the graphic carbon functionality of MXene tagged on the CdS surface. In addition to the CdS and MXene-related peaks, the new peaks at 367.5 and 392.2 cm^−1^ were assigned to the E_2g_^1^ and A_1 g_ vibration modes of MoS_2_ on the ternary composite samples. Brunauer–Emmett–Teller (BET) analysis showed that the ternary samples exhibited type IV isotherms (Fig. 3a). The ternary CdS–MXe/MoS_2_ heterostructure samples exhibited a specific surface area of 27.230 m^2^/g, which was 1.61 and 1.31 times higher than that of CdS (16.885) and CdS–MoS_2_ (20.822) samples, respectively. This could be attributed to the hierarchical surface functionality of the composite surface rather than the pristine CdS nanospheres.

XPS revealed the surface functionality and electronic states of the CdS, CdS–MXe, and CdS–MXe/MoS_2_. The survey spectrum (Fig. S9) showed the co-existence of Cd, S, Ti, C, and Mo on the composites, which agrees with the elemental mapping analysis. Figure 3b shows the high-resolution Cd 3d spectra of the CdS, CdS–MXe, and CdS–MXe/MoS_2_ samples. The binding energy peaks at 405.1 eV and 411.9 eV were associated with Cd 3d_5/2_ and Cd 3d_3/2_, respectively, which are the typical characteristics of Cd^2+^ in CdS. The function of tagging CdS with MXene and producing the MXe/MoS_2_ heterostructure with CdS resulted in a shift at the 3d_5/2_ and 3d_3/2_ positions compared to the pristine CdS. The high-resolution S 2p peaks of CdS (Fig. 3c) revealed two peak positions at 161.4 and 162.6 eV coordinated to the S 2p_3/2_ and S 2p_1/2_ of S^2−^ on the composite surface. Similar to the Cd peak position, the S 2p peaks were shifted towards the negative binding energy, resulting in a strong heterostructure interaction. The high-resolution C 1 s spectra of MXene and composite samples (Fig. 3d) were Gaussian fitted into five peaks at 281.6, 282.7, 284.6, 286.5, and 288.5 eV, representing C–Ti, C–Ti–O, C–C, C–O/C–N, and C=O, respectively [43]. The intensity of Ti–C in the ternary composites was minimized because of the encapsulation of MoS_2_ over the MXene-tagged CdS surface. The shift in the binding energy of Ti–C on the composite surface resulted in the intimate interaction of MXene with CdS, which facilitated carrier migration between the heterostructure. Figure 3e presents the high-resolution Ti 2p spectra of the MXene, CdS–MXe, and CdS–MXe/MoS_2_ samples. The three pairs of strong Ti 2p^3/2^/Ti 2p^1/2^ doublets for Ti–C (455.2/461.1 eV), Ti–O_x_ (456.6/462.2 eV), and Ti–O (458.9/463.9 eV) provide evidence of crystalline nature of MXene. The peak at 458.9 eV represents Ti–O formed by partial oxidization during the delamination process [44]. The heterostructure samples showed a lower Ti-related peak intensity, possibly due to the thin-layered surface feature and encapsulation of MoS_2_ over the MXene-tagged CdS surface. Furthermore, the high-resolution Mo 3d spectra of the composite samples (Fig. 3f) attribute the two prominent peaks at 228.4 and 231.7 eV to Mo 3d_5/2_ and Mo 3d_3/2_ of Mo^4+^, which shifted slightly to a lower binding energy compared to the pristine MoS_2_ nanoflakes. The lower binding energy shift in Mo and the higher binding energy shift in Cd compared to pristine MoS_2_ and CdS indicate an intimate contact interface between the heterostructures. A comparison of the CdS–MoS_2_ and CdS–MXe/MoS_2_ heterostructures revealed a slight shift in the S 2p and Mo 3d peak positions, resulting in a change in electronic density on the heterostructure network (Fig. S10). The actual quantity of MXene in the heterostructure composites was quantified by ICP–OES analysis. In the CdS–MXe_2.4_ sample, the MXene content in the composite sample was 2.1 wt%, which is close to the theoretical value of 2.4 wt%. Furthermore, the actual mass loading of the MXene content in the ternary composite (CdS–MXe_2.4_/MoS_2_) was 1.8 wt%, closely aligning with the initial theoretical value. The MXene wt% loss might be due to leaching or detachment from the CdS surface during the solvothermal process of growing MoS_2_ on the spherical surface.

### Photocatalytic H_2_ evolution performance

**Fig. 4:**
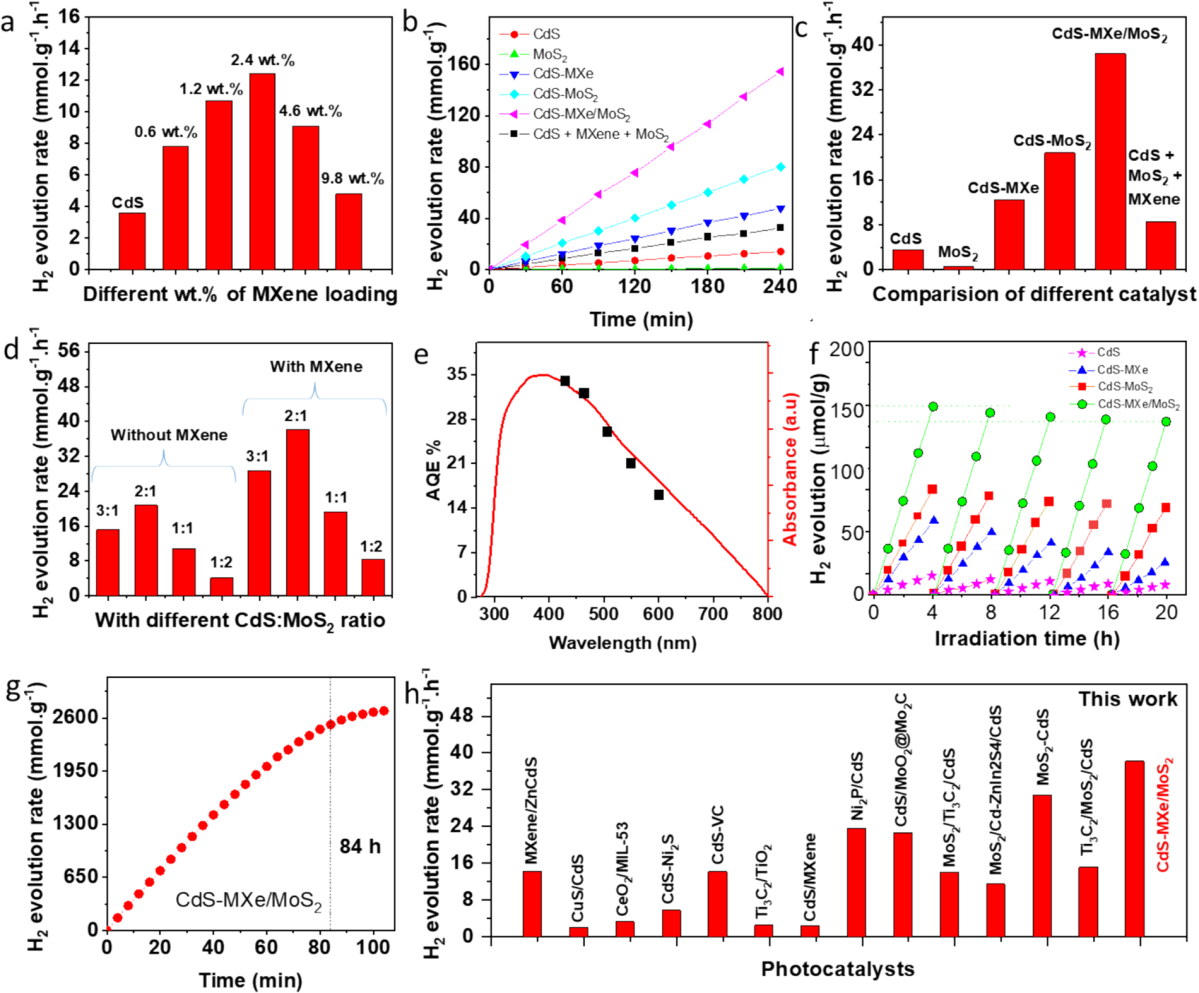
**a** Average photocatalytic activity of H_2_ production over CdS–MXe with different MXene loadings. **b** Photocatalytic H_2_ production, and **c** average photocatalytic H_2_ production rate over CdS, MoS_2_, CdS–MXe, CdS–MoS_2_, CdS–MXe/MoS_2_, and a physical mixture of CdS, MoS_2_, and MXene. **d** Photocatalytic H_2_ production over different CdS:MoS_2_ loading ratios with and without an MXene interlayer. **e** Wavelength-dependent AQY curve for H_2_ generation for CdS–MXe_2.4_/MoS_2_. **f** Cycling photocatalytic activity of binary and ternary heterostructures. **g** Time-dependent photocatalytic H_2_ production of the CdS–MXe_2.4_/MoS_2_ sample. **h** Comparison of the H_2_ evolution rate of this work and other photocatalysts reported

The photoreaction was carried out by suspending a catalyst in a lactic acid aqueous solution (10 vol%) with constant stirring, which was degassed and irradiated with a 300 W Xe lamp equipped with a 420 nm cut-off filter. The photocatalytic activity of pristine CdS with different loading densities of MXene was studied (Fig. 4a). The H_2_ evolution rate of CdS is as low as 3.6 mmol g^−1^ h^−1^, resulting from the aggregation effect and the rapid recombination of photogenerated electron–hole pairs. The surface functionalization of PDDA did not significantly affect the catalytic activity of the CdS, resulting in a similar H_2_ evolution rate (Fig. S11). Increasing the MXene loading density on the CdS from 0.6 to 2.4 wt% increased the H_2_ evolution rate from 7.8 to 12.4 mmol g^−1^ h^−1^. The result confirmed the promotion ability of MXene on the CdS surface, which increased carrier migration and minimized the photogenerated carrier recombination rate. As the MXene loading density was increased, the catalytic activity was reduced to 4.8 mmol g^−1^ h^−1^ (@ 9.8 wt% of MXene), possibly due to the light shield effect followed by the maximized carrier recombination centers. Figure 4b shows the photocatalytic H_2_ production rate of CdS, CdS–MXe_2.4_, and CdS–MXe_2.4_/MoS_2_ heterostructures, where tagging the MXene increased the H_2_ production rate and further decorating the MoS_2_ maximized the H_2_ evolution rate. Apart from MXene, decorating the MoS_2_ maximized the H_2_ evolution rate of CdS–MoS_2_ (20.8 mmol g^−1^ h^−1^) (Fig. 4c). With the advantage of in-layered MXene on the CdS–MoS_2_ heterostructure, the hierarchical CdS–MXe/MoS_2_ heterostructure resulted in a photocatalytic H_2_ production rate of 38.5 mmol g^−1^ h^−1^, which was 10.7 times higher than that of bare CdS nanospheres. Furthermore, the ternary CdS–MXe/MoS_2_ heterostructure resulted in a higher photocatalytic H_2_ production rate, which was 3.1 and 1.9 times higher than those of the CdS–MXe and CdS/MoS_2_-based binary heterostructures, respectively. The average photocatalytic activity of H_2_ production in the presence of the MXene-based inlayer was studied by controlling the growth density of MoS_2_ on CdS (Fig. 4d). On the other hand, the overloading of MoS_2_ and massive heterostructure also decreased the H_2_ evolution activity because of the lower active sites, high light shading, increased recombination centers, and destroyed core–shell morphology. The CdS:MoS_2_ ratio difference of 2:1 would be the perfect platform for high photocatalytic activity, and the inlayer effect of MXene further maximizes the catalytic activity of the hierarchical CdS–MoS_2_ heterostructure with the core–shell hierarchical assembly. The optimal MXene loading (2.4 wt%) and MoS_2_ (2:1 ratio of CdS:MoS_2_) over the CdS surface as a shell wall functionality formed a synergistic effect that significantly enhanced the photocatalytic activity compared to the bare CdS and their respective binary heterostructures. The AQY of the CdS–MXe_2.4_/MoS_2_ catalyst reached 34.6%, 32.0%, 26.3%, 21.4%, and 16.2% at the wavelengths of 420, 460, 510, 560, and 620 nm, respectively (Fig. 4e), which is higher than the pristine CdS. In addition, the photocatalytic activity of CdS–MXe_2.4_/MoS_2_ was tested under different illumination conditions (Fig. S12). Under IR irradiation, no significant activity was observed, whereas the CdS–MXe_2.4_/MoS_2_ exhibited a photocatalytic H_2_ production rate of 51.3 mmol g^−1^ h^−1^ under simulated solar light, which was 1.33 times higher than that of visible light activity under a 420 nm cut-off filter. The reusability and stability of the catalyst are the critical factors that determine its photostability for future practical applications. The photocatalytic H_2_ production rate of the CdS–MXe/MoS_2_ heterostructure showed 93.1% efficiency, which was relatively stable even after five reusable cycles, indicating the reusability and stability of the catalyst (Fig. 4f). The catalytic activity of CdS and CdS–MXe decreased by approximately 55.5 and 74.2% after five cycles, respectively. A decrease in CdS and CdS–MXe catalytic activity may be due to the poor photostability of the catalytic surface. On the other hand, fabricating the heterostructure assembly as CdS–MXe wrapped with MoS_2_ promoted the photo-corrosion stability of the CdS. Furthermore, the morphological assembly of the ternary structure before and after five reusable cycles was almost identical, resulting in the durability of the heterostructure catalyst (Fig. S13). The XRD pattern of the CdS–MXe/MoS_2_ showed no significant difference between before and after five cycles of the photocatalytic process (Fig. S14). After the reusable tests, Cd-related peaks (JCPDS No. 05–0674) were observed on the CdS and CdS–MXe samples, resulting in the reduction of Cd during the H_2_ production, which demolished the reusable properties of CdS and CdS–MXe. Nevertheless, the structural stability of the MoS_2_-based shell wall keeps the CdS from being reduced by light and helps the ternary heterostructure surface stay stable. The stability of the heterostructure was quantified through the XPS analysis for the CdS-MXe/MoS_2_ before and after the reusable photocatalytic process (Fig. S15). After the reusable process, the Cd and S-related peaks were stable, which indicate the photostability of CdS on the heterostructure surface. A red shift in the Mo-related peaks indicates that more electrons were transferred to the MoS_2_ surface, thereby promoting the catalytic activity. The catalytic reaction increased the surface oxidization rate on the MXene surface, yet the composite preserved the MXene-related interface, resulting in the ternary heterostructure interface. The XPS results were correlated with the XRD results, demonstrating that the heterostructure is stable even after the cyclic process. With the prolonged reaction time, the CdS–MXe/MoS_2_ catalyst generated H_2_ up to 90 h (Fig. 4g), and the suspension stayed similar all the time. The improved catalytic activity and stability of the CdS–MXe/MoS_2_ heterostructure can be attributed to the following: (i) the thin layered interface of metallic MXene promoted the conductivity and imparted hydrophilicity to the photocatalytic system; (ii) the efficient transfer of photogenerated holes from the CdS to MXene, facilitated by the large interfacial contact, inhibits the carrier recombination; (iii) the core–shell feature with the hierarchical surface provides numerous active sites with interior cavities, maximizing the catalytic activity; (iv) At lower active potentials, the heterostructure interface promotes electron transfer from CdS to MoS_2_, leading to the formation of H_2_ through the reaction H^+^; (v) The MXene interlayer acts synergistically with the system, inhibiting charge recombination and enhancing the activity of the CdS–MXe/MoS_2_ nanospheres through effective carrier migration. Comparing the substitution of noble metal (0.1 wt% of Pt) with CdS as a co-catalyst, the CdS–MXe catalyst resulted in 1.5 times the H_2_ production rate, indicating the role of MXene as a co-catalyst in the significant photocatalytic H_2_ evolution (Fig. S12b). Compared to the other recently reported catalyst, the CdS–MXe/MoS_2_ heterostructure showed excellent catalytic activity and stability during photocatalytic H_2_ production (Fig. 4h, Table S1).

**Fig. 5:**
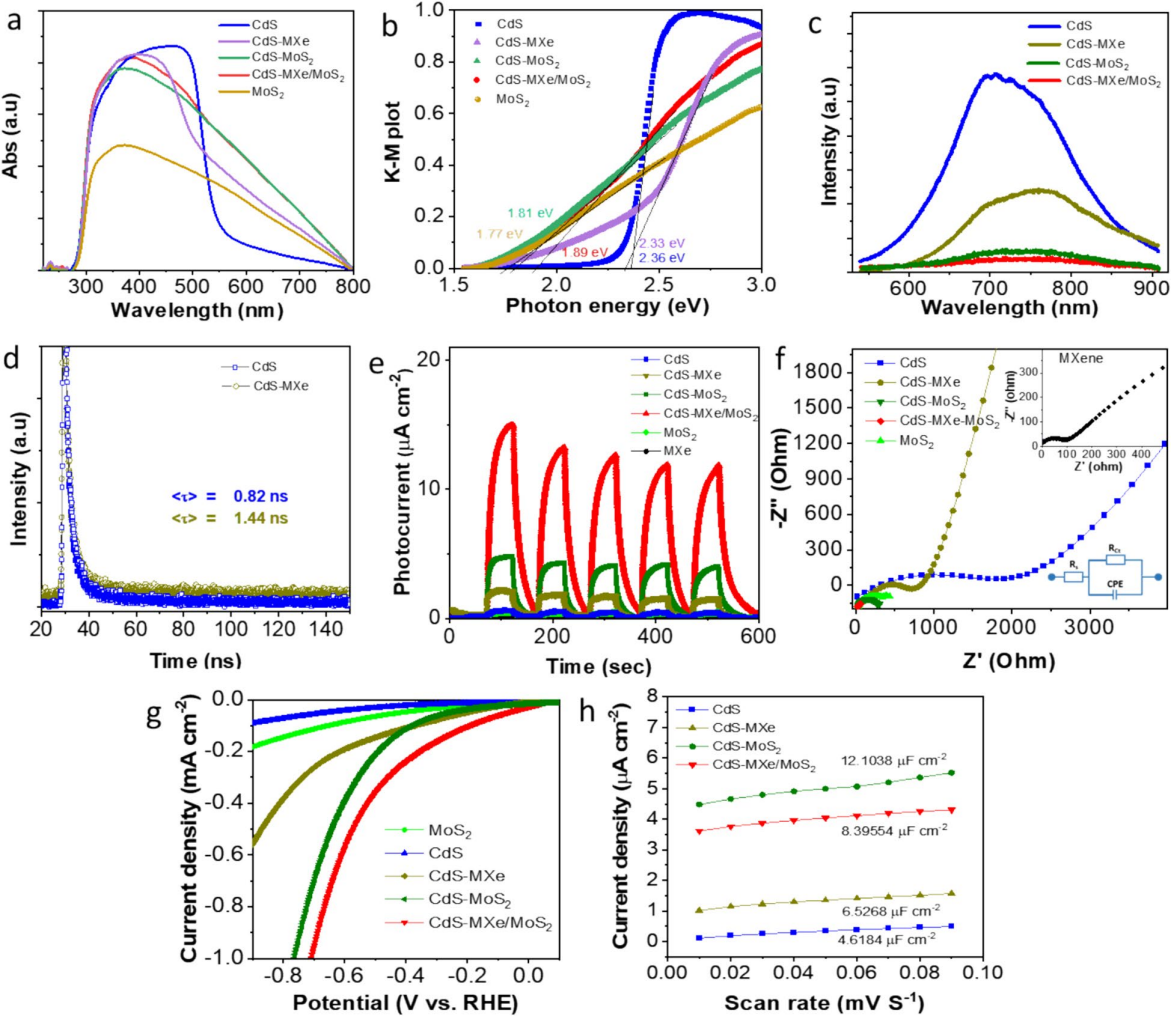
**a** UV-DRS spectra of the CdS, CdS–MXe, and CdS–MXe/MoS_2_ heterostructures. **b** K-M plot of CdS heterostructures derived from UV-DRS spectra. **c** photoluminescence, **d** TRPL spectra, **e** photocurrent response, **f** EIS Nyquist plot and the inset show the equivalent circuit diagram of the CdS–MXe/MoS_2_ electrode, **g** LSV spectra, and **h** current density differences plotted as a function of scan rates of the CdS heterostructures

The optical absorption properties of bare CdS, CdS–MXe, CdS–MoS_2_, and CdS–MXe/MoS_2_ heterostructures were studied using UV–Vis DRS (Fig. 5). The CdS nanospheres had strong visible adsorption, with the band edge adsorption at 549 nm. Tagging the MXene on the CdS surface extended the adsorption towards the visible region. Increasing the loading density of MXene on CdS resulted in higher light adsorption in the 550–800 nm region because of the background light adsorption of MXene (Fig. S16). On the other hand, the MXene-loaded CdS–MXe showed a significant red shift in the band edge adsorption, and the low loading density of MXene (0.6–4.8 wt%) resulted in high adsorption, which would be favorable for improvised photocatalytic activity. With the functionality of the MoS_2_ loading, light adsorption has been promoted because of the synergistic effect of the heterostructure assembly. According to the absorption response, the band gap of the CdS and the respective heterostructures was calculated through a K–M plot (Fig. 5b and Table S2). The band gap of the pristine CdS was determined to be 2.36 eV. Tagging the MXene on the CdS surface did not significantly alter the band gap apart from the promoted light response. Decorating the MoS_2_ on the CdS heterostructure surface, the visible response was improvised slightly because of MoS_2_ tagging, which showed a band gap of 1.81 eV. Similarly, the pristine MoS_2_ also had a band gap of 1.77 eV, indicating the narrow band gap functionality of the layered structure. Tagging CdS with MXene and MoS_2_ improved the visible response and introduced the photothermal effect that could maximize the photocatalytic activity to some extent. The improvised catalytic activity and stability were caused by effective photogenerated carrier separation and transfer capability of the catalytic surface. PL, TRPL, EIS, and LSV of the heterostructure samples were conducted to address the impact of the photocatalytic activity and quantify the carrier separation efficiency. PL is an effective tool for monitoring the separation and transfer efficiency of the photoinduced electrons, which would have a significant effect on the photocatalytic performances [45]. Under 375 nm excitation, the CdS nanospheres exhibited an intense emission peak at approximately 713 nm (Fig. 5c) owing to the band-edge emission of CdS. The PL results of the CdS–MXe/MoS_2_ exhibited a significant decrease in luminescence behavior compared to the CdS and CdS–MXe samples, confirming the inhibition of photoinduced carrier recombination. While comparing the CdS/MoS_2_ sample, the CdS–MXe/MoS_2_ sample has a lower fluorescence intensity, suggesting that the introduction of MXene influenced the inhibition of carrier recombination through the effective separation of photocarriers. The heterostructure interface of MXe/MoS_2_-based dual surface functionality on CdS increased the charge transfer kinetics on CdS, suppressing photogenerated carrier recombination through effective charge carrier separation and promoting photocatalytic H_2_ production efficiency. The TRPL experiments (Fig. 5d) showed that the tagging of MXene with CdS results in a longer average lifetime (τ = 1.44 ns) than the CdS (τ = 0.82 ns). The CdS–MXe catalyst exhibited (Table. S3) significant inhibition in charge nonradiative recombination (τ1) and faster interband charge transfer (τ2) that was promoted through interfacial transfer at the interface, which increased the lifetime of photogenerated charge carriers. The presence of MXene as an interlayer effectively improved the hole transport efficiency, inhibited carrier recombination, and prolonged the carrier life. The transition photocurrent responses of the CdS, CdS–MXe, CdS–MoS_2_, and CdS–MXe/MoS_2_ samples were analyzed (Fig. 5e) to understand the photostability and photocarrier responses. The CdS–MXe/MoS_2_ samples exhibited a much stronger photocurrent response (11.89 μA cm^−2^) than the bare CdS and binary composites, indicating rapid transfer and separation of photocarriers and limited recombination. EIS was used to quantify the interfacial carrier recombination and charge transfer ability of the catalyst (Fig. 5f) and fitted by an equivalent circuit (inset of Fig. 5f). EIS (Fig. 5f) of the CdS–MXe/MoS_2_ catalysts revealed a smaller arc radius (low charge transfer resistance) than the CdS, CdS–MXe, and CdS–MoS_2_, indicating improvised internal charge transfer at the ternary interface. The Nyquist plot resulted in minimal internal transfer resistance for the CdS–MXe/MoS_2_ (273 Ω), maximizing the effective transportation of charge carriers. Photocatalytic H_2_ production depends mainly on the hydrogen evolution reaction (HER) overpotential during the catalytic reaction. The LSV was conducted (Fig. 5g) with Ag/AgCl and Pt foil as the reference and counter electrodes, respectively, to examine the ability of the catalyst to reduce the photons to H_2_. The CdS (0.031 mA cm^−2^) and MoS_2_ (0.065 mA cm^−2^) had the lowest cathodic photocurrent density, indicating a poor H_2_ evolution reaction on their surfaces. Compared to the pristine network, MXene and MoS_2_-loaded CdS showed a considerable increase in the cathodic current (0.142 mA cm^−2^ and 0.210 mA cm^−2^, respectively), indicating the improvised carrier separation followed through the heterostructure interface. In addition, the ternary interface of CdS–MXe/MoS_2_ exhibited a cathodic current density of 0.356 mA cm^−2^ at a similar potential of − 0.5 V vs. RHE at pH 5.5. The higher cathodic current density of CdS–MXe/MoS_2_ was approximately 11.5 and 5.5 times higher than that of CdS and MoS_2_, respectively. The channeling effect between the heterostructure interfaces accelerated the rapid interfacial electron transfer, which is more favorable and suitable for H_2_ production under photoirradiation. The electrochemical active surface area (ECSA) of the catalyst also confirmed its high catalytic ability. The ECSA was estimated using the double-layer capacitance (C_dl_) method according to the CV method within a non-Faradaic region at different scan rates. The current density vs. scan rate was plotted (Fig. 5h) against a certain potential window (0.10 V vs. Ag/AgCl to 0.30 V vs. Ag/AgCl), yielding a straight line with a slope equaling twice the C_dl_, which equals ECSA (Fig. S17). The ECSA value of CdS–MXe/MoS_2_ was 12.10 μF cm^−2^, which is higher than CdS (4.61 μF cm^−2^), CdS–MXe (6.53 μF cm^−2^), and CdS–MoS_2_ (8.39 μF cm^−2^). Upon normalizing the LSV curve with ECSA, the ternary CdS–MXe/MoS_2_ catalyst still resulted in higher hydrogen evolution reaction (HER) activity (Fig. S17e). This evidence shows that the improvised H_2_ evolution was elevated through the ECSA, and enhanced intrinsic activity of the catalyst resulted from its electronic structure. A comparison of the current density of CdS–MXe/MoS_2_ with that of CdS–MoS_2_ showed that the effective charge migration through MXene and the strong bonding of S atoms to H^+^ on the MoS_2_ surface would have the synergistic effect that accelerated the H_2_ evolution reaction.

**Fig. 6:**
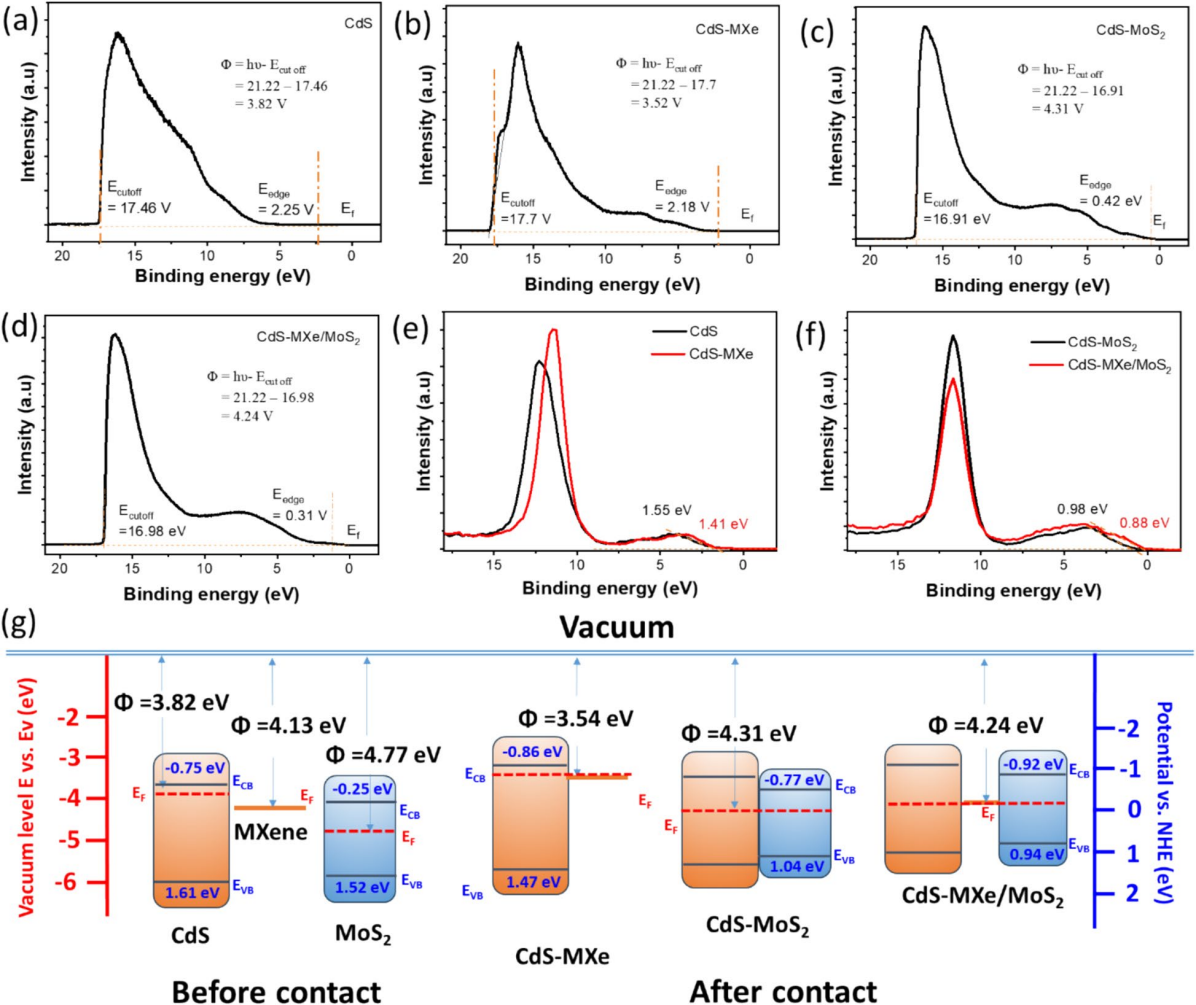
UPS spectra of the **a** CdS, **b** CdS–MXe, **c** CdS–MoS_2_, and **d** CdS–MXe/MoS_2_. XPS Valanced spectra of **e** CdS and CdS–MXe, **f** CdS–MXe, and CdS–MXe/MoS_2_. **g** Schematic diagram of CdS, CdS–MXe, CdS–MoS_2_ and CdS–MXe/MoS_2_ band position

Mott‒Schottky analyses were conducted to understand the donor density and flat band potential (Fig. S18). The positive slope of CdS and MoS_2_ addressed the n-type semiconducting properties, and binary and ternary heterostructures also resulted in similar semiconducting characteristics. The slope of the Mott–Schottky was related to the donor density (N_d_) of the catalyst; a smaller slope indicated a larger N_d_ [38]. The calculated slope of the tangent line in the Mott‒Schottky plot of the CdS, CdS–MXe, CdS–MoS_2_, and CdS–MXe/MoS_2_ nanospheres (Fig. S18, 500 Hz) was 5.078 × 10^12^ cm^−3^, 2.946 × 10^14^ cm^−3^, 2.652 × 10^18^ cm^−3^ and 2.064 × 10^18^ cm^−3^, respectively. The heterostructures had a higher N_d_ than the pristine CdS, indicating enhanced photoelectric properties. The N_d_ of CdS–MXe/MoS_2_ is nearly 1.28 times higher than that of CdS–MXe, resulting from the accumulation of more charge carriers beneficial for hydrogen evolution. The in-layered metallic characteristic and the hierarchical heterostructure assembly provide evidence of a higher charge density at the Fermi energy level than that of CdS and CdS–MoS_2_. Based on theoretical predictions, the MXene interface promoted the high aggregated electron density on the MoS_2_ surface compared to the binary contact interface of CdS–MoS_2_ [36]. The calculated flat band potentials of CdS, MXene, and MoS_2_ were − 0.47, − 0.13, and 0.04 V (vs. NHE), respectively. The fabricated heterostructures exhibited a flat band potential of − 0.196 and − 0.152 V (Vs. NHE) for the CdS–MoS_2_ and CdS–MXe/MoS_2_, respectively, which is more negative than pristine MoS_2_. The band structure of the heterostructures met the reduction potential for H_2_ production, which expresses the influence of the photocatalytic activity (Fig. 6).

The UPS was used to identify the Fermi level, Fermi edge, and the work function of the CdS and composite heterostructures. The work function (Φ) was calculated using the following equation: Φ = hυ – E_cutoff_ [38], where hυ is the incident photon energy (21.22 eV) and E_cutoff_ is the cut-off binding energy at the cut-off edge. The calculated work functions of CdS, CdS–MXe, CdS/MoS_2_, and CdS–MXe/MoS_2_ were 3.82, 3.54, 4.31, and 4.24 eV (vs. vacuum), respectively (Fig. 6a–d and Fig. S19). Following the heterostructure contact, the different E_f_ effectively produced a built-in electric space charge layer. With band alignment, CdS–MXe and CdS/MoS_2_ would form a Schottky heterostructure and a type-I heterostructure, respectively, because of the potential differences between the different materials. Figure 6e, f shows the XPS valence-band spectra to quantify the position. The calculated VB potentials of CdS, CdS–MXe, CdS–MoS_2_, and CdS–MXe/MoS_2_ were 1.61, 1.47, 1.04, and 0.94 eV, respectively, based on the equation (E_NHE_ = Φ + X ‒ 4.44) [46]. E_NHE_ is the potential of the normal hydrogen potential, Φ = 4.5 eV is the work function of the analyzer, and X is the cut-off of the VB band edge position. The calculated band gap of the CdS, CdS–MoS_2_, and CdS–MXe/MoS_2_ was 2.36, 2.33, 1.81, and 1.89 eV, respectively, which was derived from the K–M plot (Fig. 5b) coupled with the equation (E_CB_ = E_VB_–E_g_), and the conduction band position of the CdS, CdS–MXe, CdS/MoS_2_, and CdS–MXe/MoS_2_ was calculated as − 0.75, − 0.86, − 0.77, and − 0.92 eV vs. NHE, respectively. The schematic band positions in Fig. 6g suggest the photocatalytic mechanism of the ternary heterostructure. Because CdS has a higher Fermi energy level because of its lower work potential than MXene and MoS_2_, which promotes electron transfer from CdS to MXene and MoS_2_ even before contact. After the heterostructure formation, the fermi energy level was shifted to reach equilibrium. In addition, the conduction band position of CdS was down-bent on the heterostructure interface, which suppressed the electron backflow and promoted the elective separation of photogenerated charge carriers that facilitate a high photocatalytic H_2_ production rate.

**Scheme 2:**
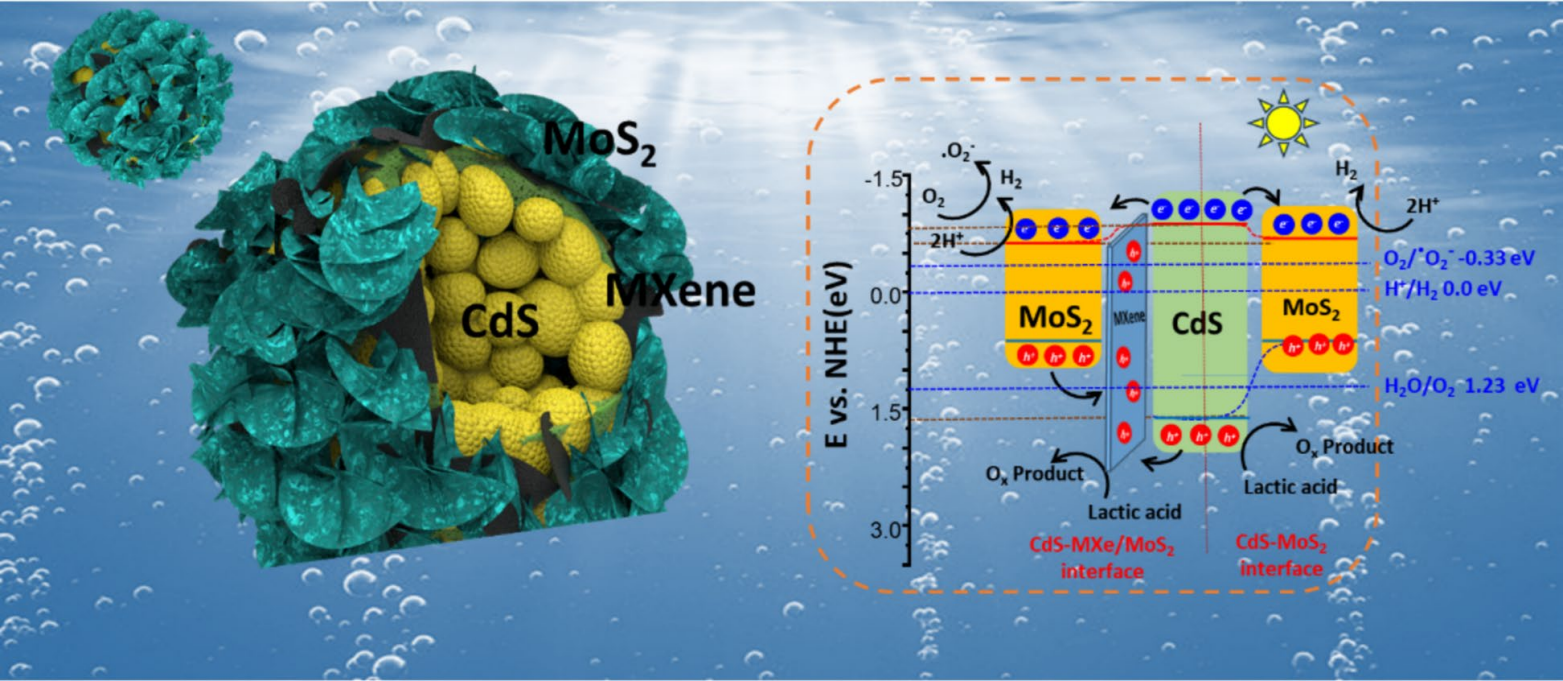
Schematic diagram of the photocatalytic mechanism of the ternary CdS–MXe/MoS_2_ heterostructure

The charge transfer mechanism of the photocatalytic H_2_ production on CdS–MXe/MoS_2_ heterostructures was proposed based on the experimental (XPS and UPS) information and the photocatalytic activity (Scheme 2). Under light irradiation, the photogenerated electrons were excited from the VB to the CB of CdS, where they migrated to the layered MXene and spontaneously transferred to MoS_2_, which reduced the adsorbed H^+^ to H_2_. As an effective hole migrator, MXene would drag the holes from the CdS and MoS_2_ surfaces and inhibit photogenerated carrier recombination. The photogenerated holes oxidized the lactic acid on its surface, which was used as a sacrificial agent. The photogenerated electrons at CdS recombined quickly in the absence of a heterostructure interface, which led to low catalytic activity. The intimidating contact interface of layered MXene with the metallic characteristic provides a short pathway to separate the photogenerated carriers because of the convergence of the fermi level with restricted backflow of electrons, which inhibits the electron–hole pair recombination and facilitates photocatalytic H_2_ production. The effective electron transport from CdS to MoS_2_ forms an electron-rich environment on the plane of MoS_2_, which reduces the adsorbed H^+^ to H_2_. The hierarchical, vertically aligned MoS_2_ induces the beneficial edge active sites that enhance the photocatalytic activity. The superior photocatalytic activity of hierarchical CdS–MXe/MoS_2_ nanospheres for Pt-free H_2_ production may be due to the following reasons: (i) the feasible hierarchical core–shell assembly with the in-layered MXene promoted efficient carrier separation with inhibited carrier recombination; (ii) photocarriers were shuttled through MXene that produce a high carrier density on the MoS_2_ that maximizes H_2_ production; (iii) producing a lower activation potential with more active sites with co-catalytic functionality offers the platform for the effective reduction of H^+^ for H_2_ production. Furthermore, the pristine CdS trend was agglomerated and influenced easily by photocorrosion through the rapid reduction of Cd and S. While tagging, MXene promoted charge migration, and MoS_2_ protected the CdS surface functionality by obscuring the photocorrosion. As a result, photogenerated carrier recombination was minimized by effective carrier separation. Through the synergistic effect of the ternary CdS–MXe/MoS_2_ heterostructure assembly, the photocorrosion was effectively avoided, and the carrier separation efficiency was improved, resulting in prodigious photocatalytic activity and noteworthy catalytic stability.

## Conclusion

As a ternary core–shell system, the solution-assisted CdS nanospheres were tagged with MXene through an electrostatic process, and they grew MoS_2_ nanoflakes through a solvothermal process to construct an intimate CdS–MXe/MoS_2_ heterostructure interface. The characterization details confirmed the successful formation of ternary heterostructure assemblies, similar to the hierarchical core–shell structural interface. This unique structural feature can overcome the inherent limitations of CdS and promote carrier separation efficiency and visible photocatalytic H_2_ production. The ternary CdS–MXe/MoS_2_ heterostructure catalyst exhibited a photocatalytic H_2_ production rate of 38.5 mmol g^−1^ h^−1^, which was 10.7, 3.1, and 1.9 times higher than those of CdS, CdS–MXe, and CdS/MoS_2_, respectively, under visible light irradiation and exhibited superior stability even after five reuse cycles. The optical, UPS, and XPS analyses confirmed that the composed ternary interface of CdS–MXe/MoS_2_ enhanced the intimate contact, enhanced the carrier density, and maximized the photocatalytic H_2_ production. The convergence fermi level and limited backflow of electrons on the heterostructure interface inhibited the carrier recombination rate and boosted the photocatalytic H_2_ production. The heterostructure assembly inhibited the photocorrosion of CdS and promoted the photostability; the heterostructure assembly of CdS/MoS_2_ with the ultra-thin layered MXene interface helped bridge the translation gap to promote the photocatalytic activity and durability of the catalyst. This study provides a new avenue for developing a low-cost ternary heterostructure photocatalyst for water splitting in practical use.

## Supplementary Information

Supplementary Material 1.

## Data Availability

The datasets used and analyzed during the current study are available from the corresponding author upon reasonable request.
