# Correction: Interfacial charge transfer on hierarchical synergistic shell wall of MXene/MoS_2_ on CdS nanospheres: heterostructure integrity for visible light responsive photocatalytic H_2_ evolution

**DOI:** 10.1186/s40580-024-00469-8

**Published:** 2025-01-27

**Authors:** Kugalur Shanmugam Ranjith, Ali Mohammadi, Ganji Seeta Rama Raju, Yun Suk Huh, Young-Kyu Han

**Affiliations:** 1 Department of Energy and Material Engineering, Dongguk University-Seoul, Seoul, 04620 South Korea; 2 Department of Biological Sciences and Bioengineering, Nano Bio High-Tech Materials Research Center, Inha University, Incheon, 22212 South Korea

**Correction to: Nano Convergence (2024) 11: 51**

10.1186/s40580-024-00454-1

The original publication of this article [1] contained incorrect project number in Acknowledgement section. The correct project number should be “RS-2024-00406723” instead of “2022R1C1C1010601”.

The incorrect and correct Acknowledgement statement are listed with this correction article. The original article has been updated.

Incorrect:

**Acknowledgements**

This work was supported by the National Research Foundation of Korea (NRF) grant funded by the Korean Government (MSIT) (2022M3J7A1062940, 2022R1C1C1010601, 2022R1A2C2008968).

Correct:

**Acknowledgements**

This work was supported by the National Research Foundation of Korea (NRF) grant funded by the Korean Government (MSIT) (2022M3J7A1062940, RS-2024-00406723, 2022R1A2C2008968).
