# Supplementary material for: Interfacial charge transfer on hierarchical synergistic shell wall of MXene/MoS2 on CdS nanospheres: heterostructure integrity for visible light responsive photocatalytic H2 evolution
Source: Nano Converg. 2024 Dec 2;11:51. doi: 10.1186/s40580-024-00454-1 (PMC11612105; doi:10.1186/s40580-024-00454-1)
Supplement: Supplementary file 1 — Supplementary Material 1. [file 40580_2024_454_MOESM1_ESM.docx]

**Interfacial charge transfer on hierarchical synergistic shell wall of MXene/MoS_2_ on CdS nanospheres: Heterostructure integrity for visible light responsive photocatalytic H_2_ evolution**

# *Kugalur Shanmugam Ranjith^a^,* *Ali Mohammadi^a^, Ganji Seeta Rama Raju^a^**, Yun Suk Huh^b,*^, Young-Kyu Han^a,*^*

# ^a^Department of Energy and Material Engineering, Dongguk University-Seoul, Seoul 04620, South Korea

# ^b^Department of Biological Engineering, Inha University, Incheon 22212, South Korea

^*^Corresponding authors: yunsuk.huh@inha.ac.kr (Y. S. Huh), ykenergy@dongguk.edu (Y.-K. Han)

**
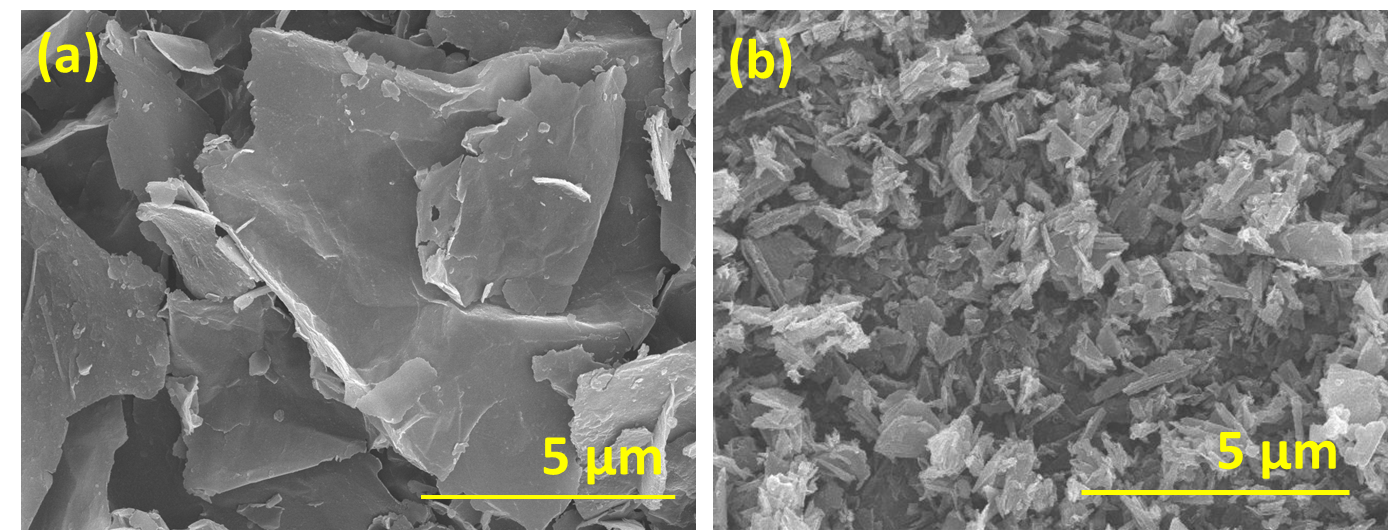
**

**Fig. S1.** SEM image of as-prepared (a) MXene and (b) delaminated MXene nanosheets.


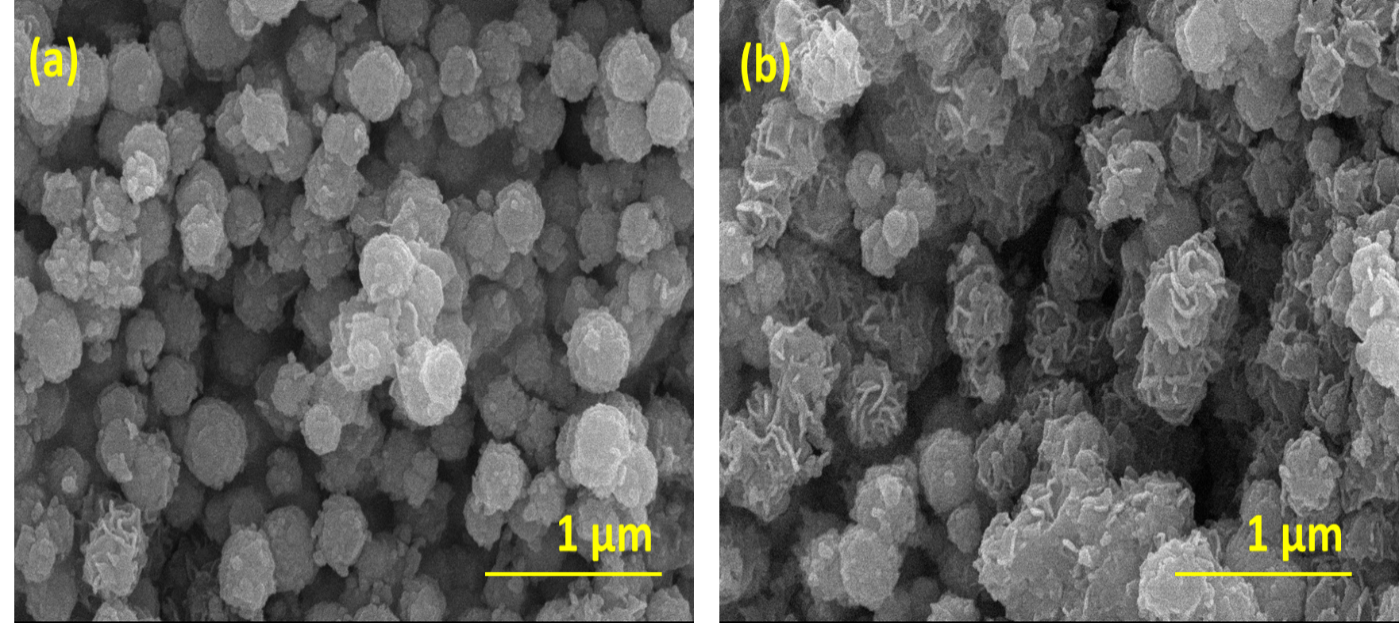


**Fig. S2.** SEM image of (a) CdS-MoS_2_ and (b) CdS-MXe/MoS_2_ heterostructures.

**
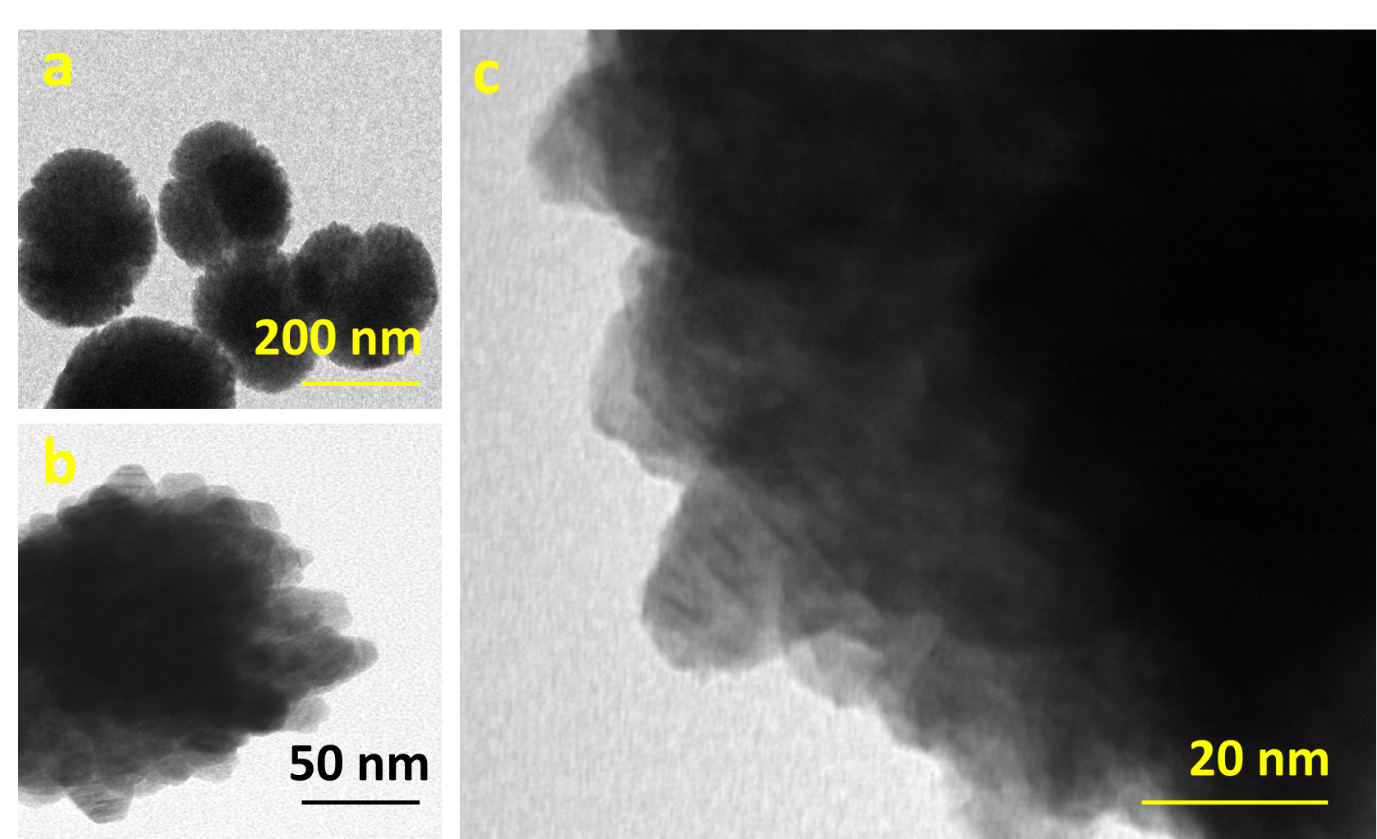
**

**Fig. S3.** TEM images of the pristine CdS nanospheres.


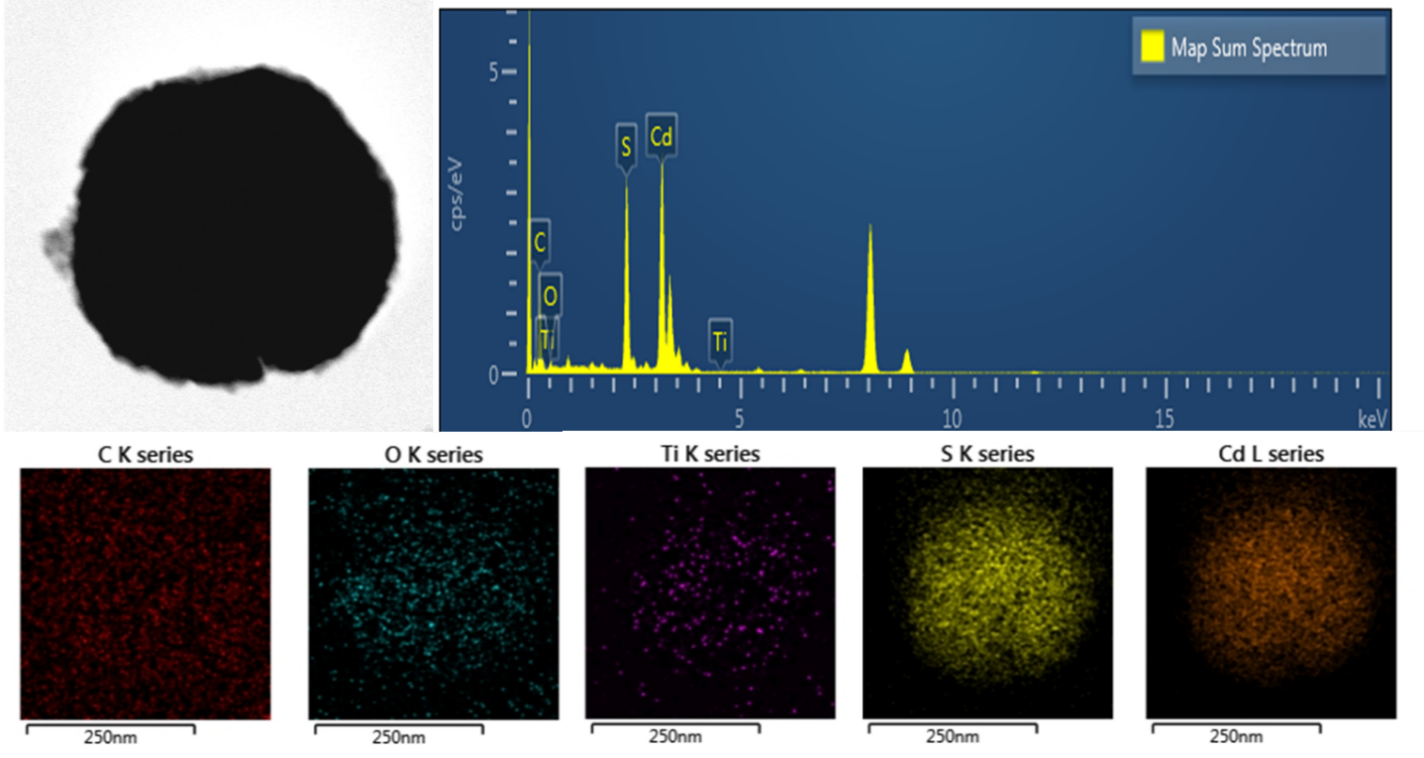


**Fig. S4.** EDAX spectra and mapping images of CdS-MXe_2.4_ nanostructures.

**
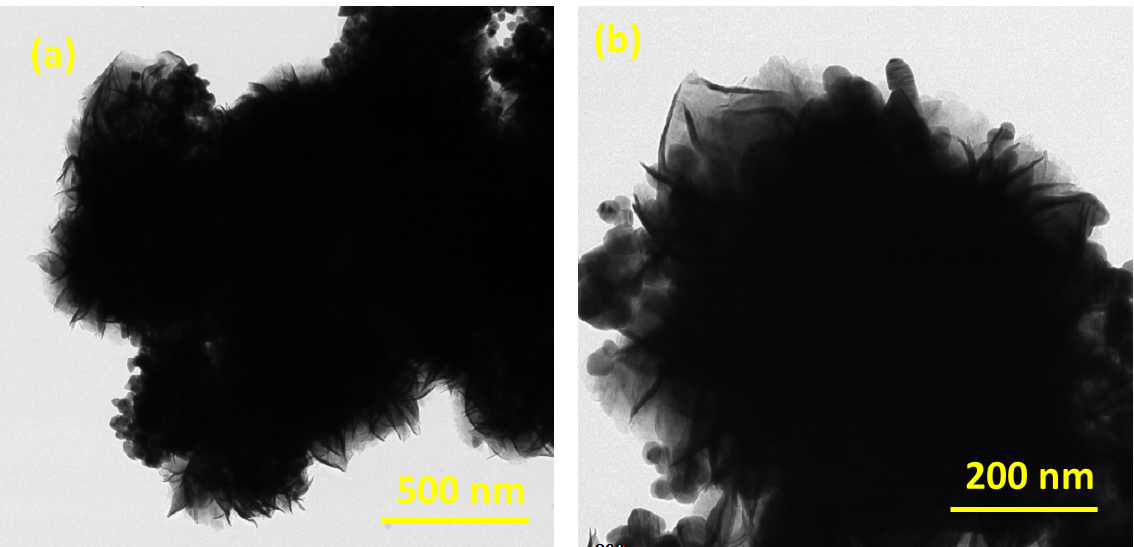
**

**Fig. S5.** TEM of the CdS-MoS_2_ heterostructure nanospheres.

**Fig. S6.** XRD spectrum of the MAX, MXene, and delaminated MXene nanostructures.

**
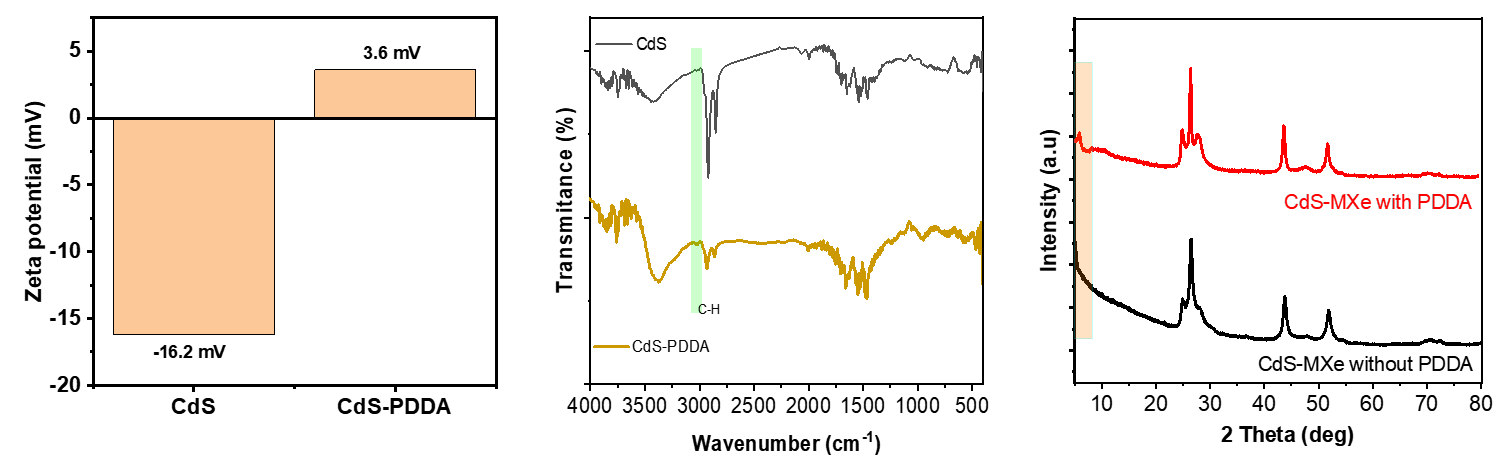
**

**Fig. S7.** (a) Zeta potential and (b) FTIR spectra of the CdS and CdS-PDDA. (c) XRD spectrum of the PDDA functionalized and non-functionalized CdS-MXe nanostructures.


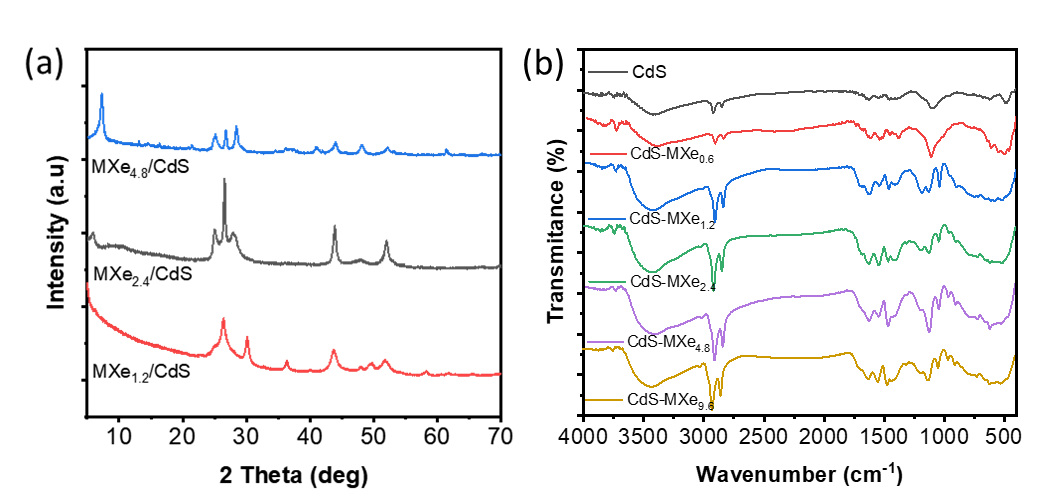


**Fig. S8.** (a) XRD spectrum of CdS/MoS_2_ with different loading densities of MXene as interlayer and (b) FTIR spectrum of the CdS with different loading densities of MXene.

**Fig. S9.** XPS survey spectrum of the CdS, CdS-MXe, CdS-MoS_2_, CdS-MXe/MoS_2_ heterostructures.

**
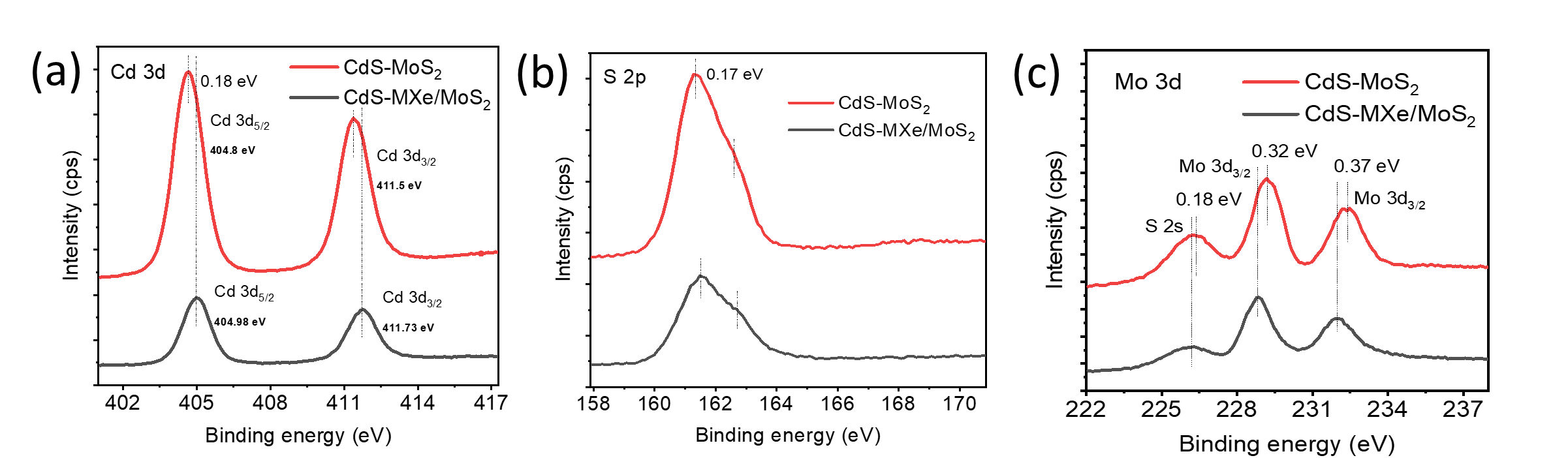
**

**Fig. S10.** High-resolution XPS spectra of (a) Cd 3d, (b) S 2p, and (c) Mo 3d for the CdS-MoS_2_ and CdS-MXe/MoS_2_ samples.

**Fig. S11.** Photocatalytic H_2_ evolution for the PDDA functionalized and non-functionalized CdS catalyst under visible irradiation with 420 nm cutoff filter.


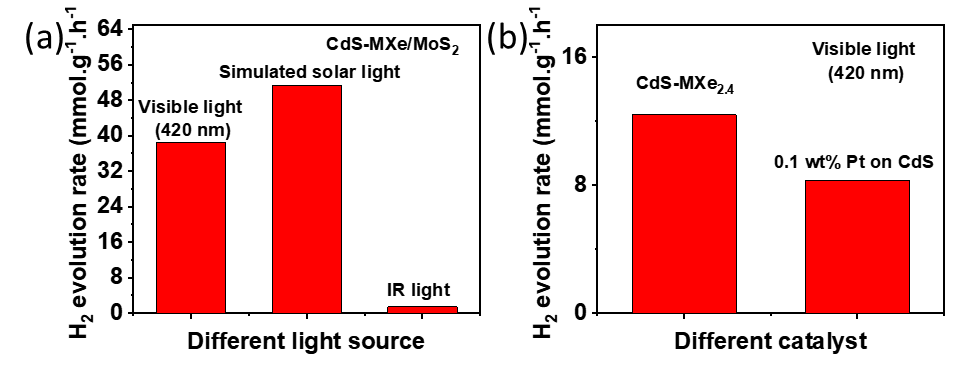


**Fig. S12.** (a) Photocatalytic H_2_ evolution for the CdS-MXe_2.4_/MoS_2_ catalyst under different light irradiation. (b) Photocatalytic H_2_ evolution for the 0.1 wt.% Pt loaded CdS catalyst under visible irradiation with 420 nm cutoff filter.

**
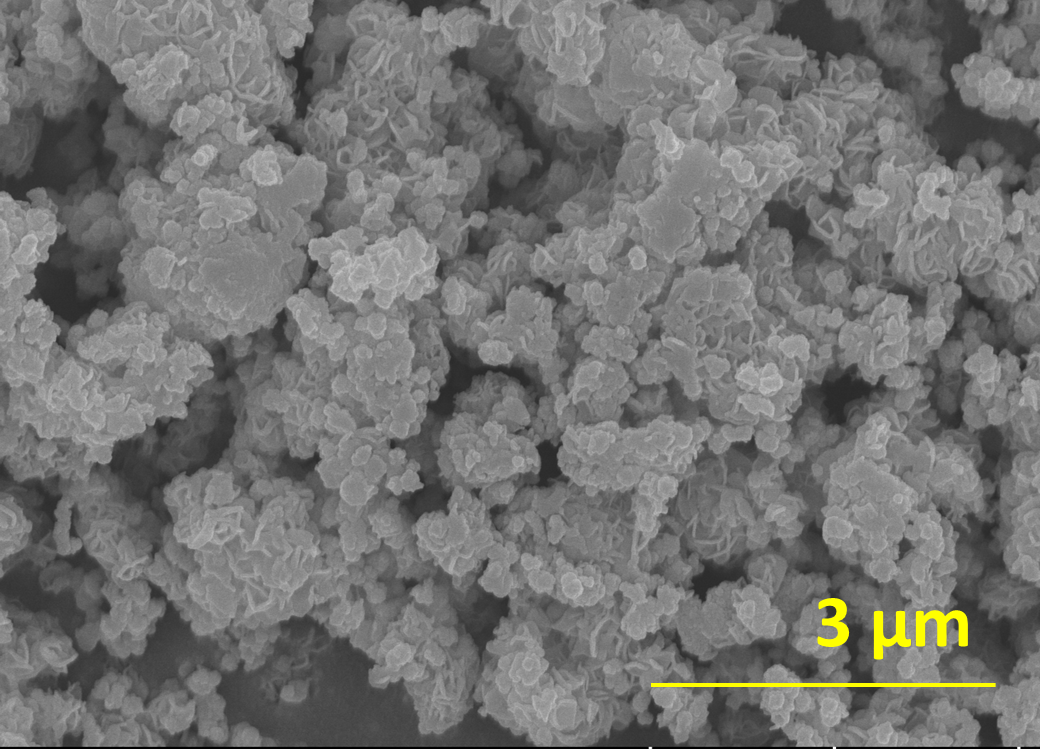
**

**Fig. S13**. SEM image of the CdS-MXe_2.4_/MoS_2_ catalyst after five reusable catalytic cycles.

**Fig. S14**. XRD spectrum of CdS, CdS-MXe_2.4_, and CdS-MXe_2.4_/MoS_2_ catalyst after five reusable catalytic cycles.

**
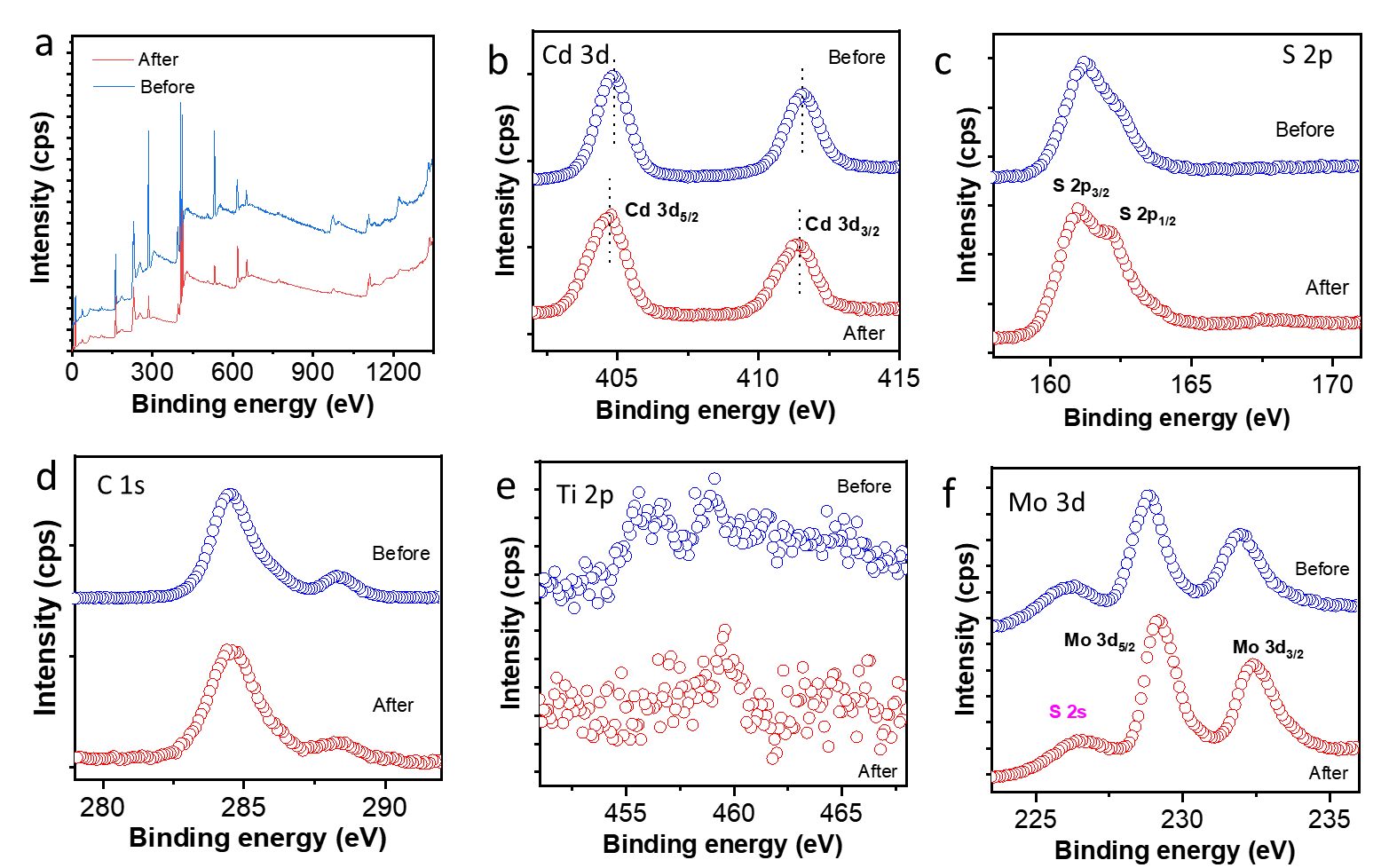
**

**Fig. S15**. XPS spectrum (a) Survey, (b) C 1s, (c) Cd 3d, (d) S 2p, (e) Ti 2p, (f) Mo 3d of the CdS-MXe_2.4_/MoS_2_ catalyst before and after five reusable catalytic cycles.

**Table S1.** Comparing the photocatalytic H_2_ evolution activity with other reported photocatalysts

| **Photocatalysts** | **Maximum rates**  **(mmol h^−1^ g^−1^)** | **Scavengers** | **Light source (Xe lamp)** | **420 nm AQE (%)** | **References** |
| --- | --- | --- | --- | --- | --- |
| CdS-MXe/MoS_2_ nanospheres | 38.5 | 10% lactic acid | λ > 420 nm | 34.6 | This work |
| CdS-MoS_2_ nanospheres | 20.8 | 10% lactic acid | λ > 420 nm | -- | This work |
| MXene/ZnCdS | 14.17 |  | λ > 420 nm |  | [1] |
| CuS/CdS | 2.076 | 2 vol% lactic acid | λ > 420 nm | 17.2 | [2] |
| CeO_2_/MIL-53 | 3.286 | 10 vol% methanol | Visible light | --- | [3] |
| CdS-Ni_2_S | 18.96 | Na_2_S-Na_2_SO_3_ | Visible light | 4.8 | [4] |
| CdS-VC | 14.2 | 10% lactic acid | λ > 420 nm | 8.7 | [5] |
| PtO-Ti_3_C_2_/TiO_2_ | 2.54 | 50% methanol | Visible light | 4.2 | [[6]](https://doi.org/10.1016/j.cej.2021.129695) |
| CdS/MXene | 2.407 | 10 vol% lactic acid | λ > 420 nm | 35.6 | [[7]](https://doi.org/10.1016/j.apcatb.2019.118382) |
| Ni_2_P/CdS | 1.18 | 10 vol% lactic acid | λ > 400 nm | 56 | [[8]](https://doi.org/10.1016/j.apcatb.2020.119443) |
| CdS/MoO_2_@Mo_2_C | 22.672 | 20 vol% lactic acid | λ > 420 nm | --- | [9] |
| MoS_2_/Ti_3_C_2_/CdS | 14.1 | 10% lactic acid | Simulated sunlight |  | [[10]](https://doi.org/10.1016/j.colsurfa.2022.129746) |
| MoS_2_/Cd-ZnIn_2_S_4_/CdS | 11.49 | 20% lactic acid | λ > 420 nm | 6.17 | [[11]](https://doi.org/10.1002/sstr.202300569) |
| MoS_2_-CdS | 30.83 | Na_2_S-Na_2_SO_3_ | λ > 420 nm | 19 | [[12](https://doi.org/10.1016/j.cej.2019.122053)] |
| Ti_3_C_2_/MoS_2_/CdS | 15.2 | 27% lactic acid | λ > 420 nm | 42.1 | [[13]](https://doi.org/10.1016/j.ijhydene.2021.12.180) |

**Fig. S16**. UV-DRS spectra of the CdS-MXe catalyst with the function of increasing the loading densities of MXene.

**Table S2.** Adsorption edge and Band gaps of the CdS, CdS-MXe, CdS-MoS_2_, and CdS-MXe/MoS_2_ nanospheres

| **Samples** | **Adsorption edge (nm)** | **Band gap (eV)** |
| --- | --- | --- |
| **CdS** | 548.5 | 2.36 |
| **MoS_2_** | ⁓800 | 1.77 |
| **CdS-MXe_2.4_** | 578.4 | 2.33 |
| **CdS-MoS_2_** | ⁓800 | 1.81 |
| **CdS-MXe/MoS_2_** | ⁓800 | 1.87 |

**Table S3.** The summary of Lifetime PL measurements

| **Samples** | τ_1_ | A | B_1_ | τ_2_ | B_2_ | τ_ave_ |
| --- | --- | --- | --- | --- | --- | --- |
| **CdS** | 2.0649E-9 sec | 4.2124 | 0.0327 | 2.4505E-10 sec | 0.1390 | 0.82286E-9 sec |
| **CdS-MXe_2.4_** | 8.2595E-10 sec | 22.2821 | 0.0771 | 5.4-22E-9 sec | 0.0197 | 1.4412E-9 sec |

**Notes:**

The average PL emission lifetime (τ_ave_) could be calculated according to the following formula:

$$\tau_{ave} =\frac{B\tau_{\boldsymbol{1}}^{\boldsymbol{2}}\mathbf{+}B_{2}\tau_{\boldsymbol{2}}^{\boldsymbol{2}}}{B_{1}\tau_{1}\mathbf{+}B_{2}\tau_{2}}$$

**
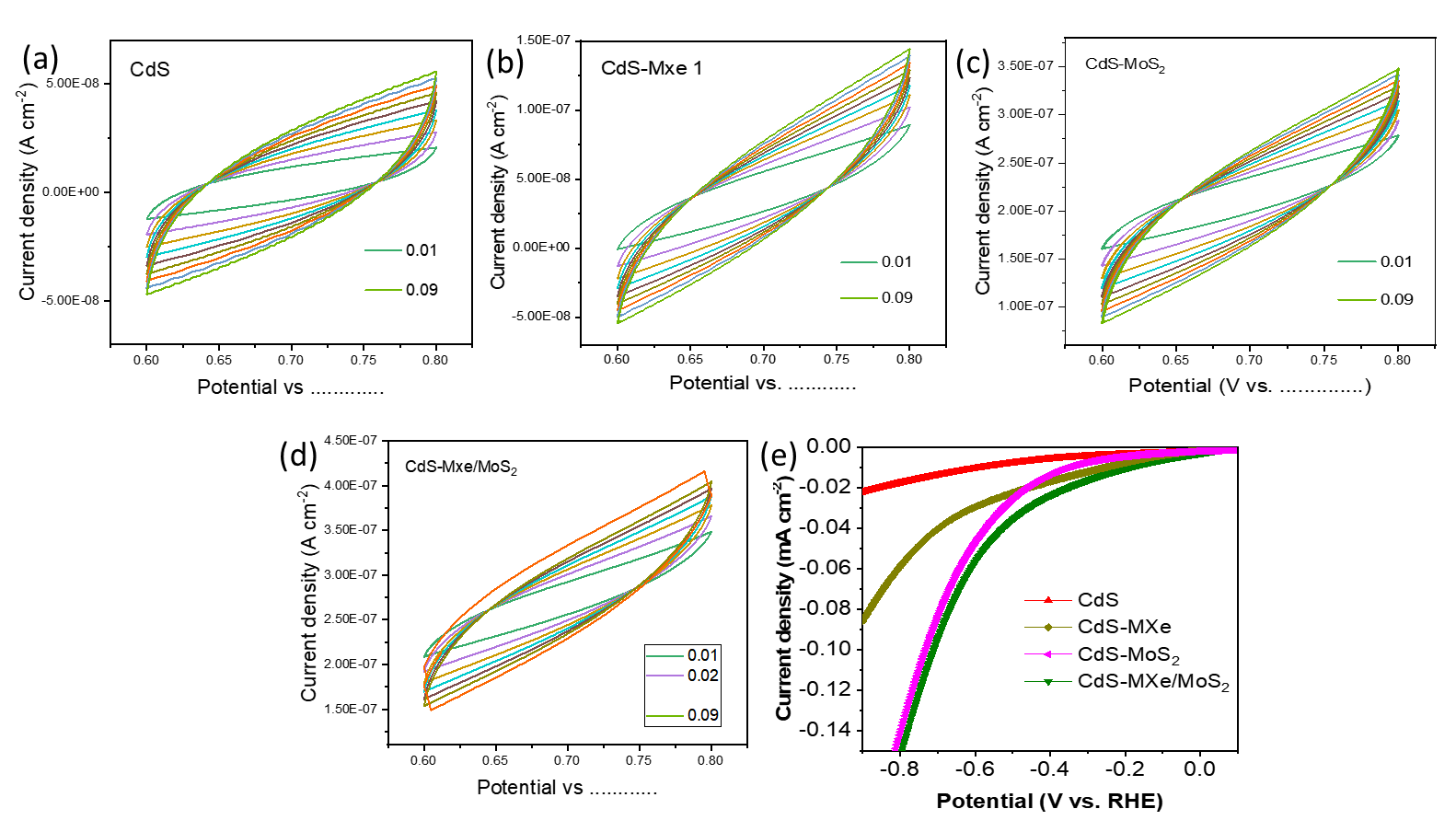
**

**Fig. S17**. Electrochemical surface area calculation of the CdS, CdS-MXe, CdS-MoS_2_ and CdS-MXe/MoS_2_. (e) Normalized LSV curve with the ECSA for the respective catalyst.

**
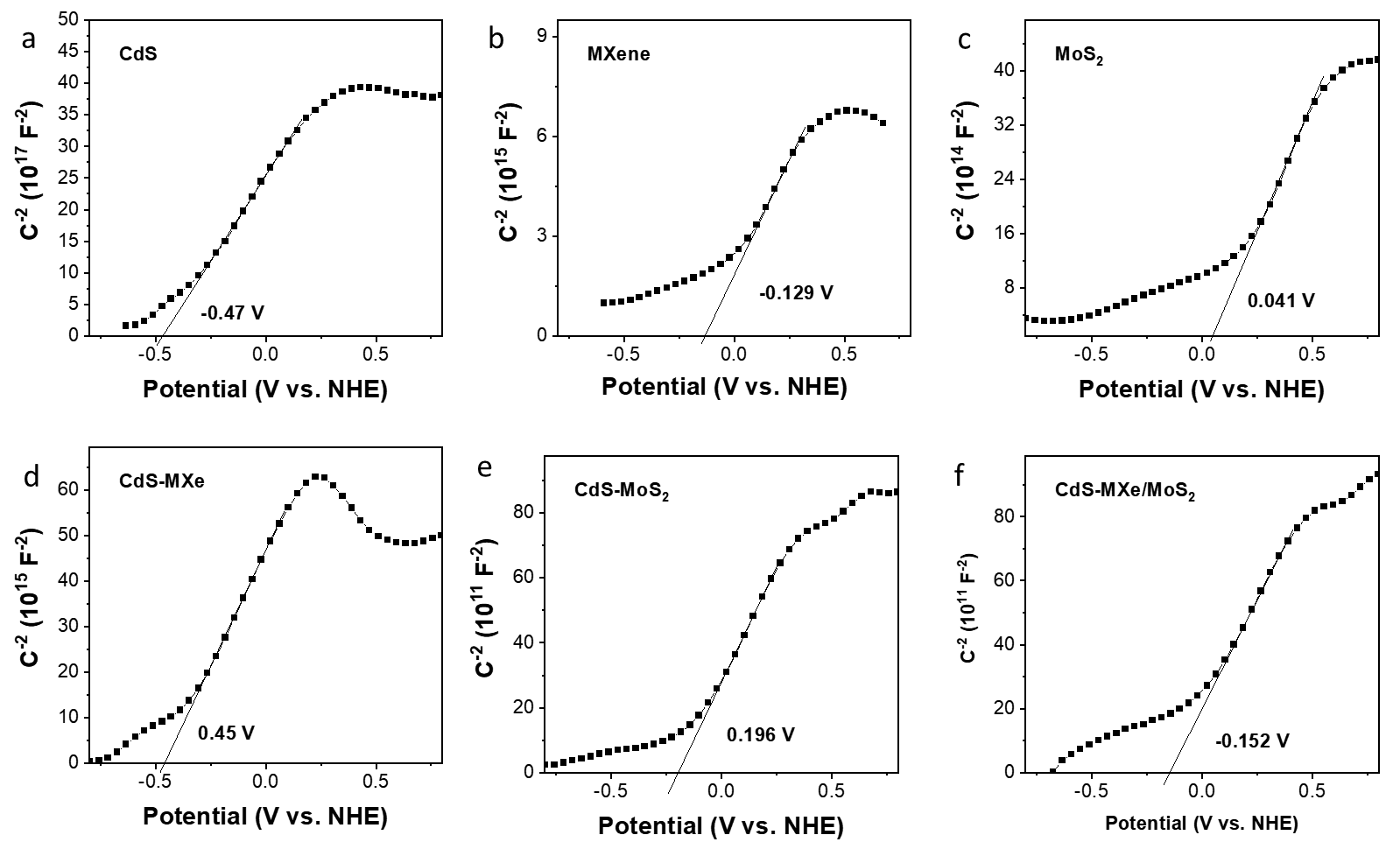
**

**Fig. S18**. Mott Schottky analysis of the CdS, MXene, MoS_2_, CdS-MXe, CdS-MoS_2_ and CdS-MXe/MoS_2_.

**
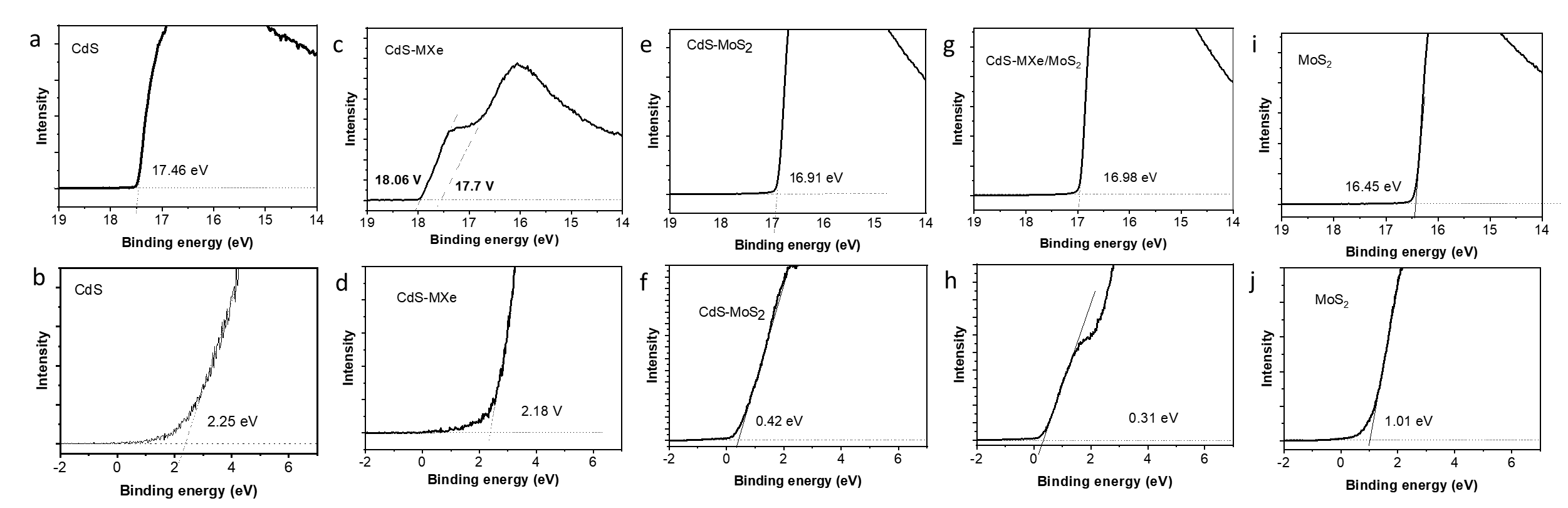
**

**Fig. S19**. UPS spectra of the different samples. Cut off edge of (a) CdS, (c) CdS-MXe, (e) CdS-MoS_2_, (g) CdS-MXe/MoS_2_ and (i) MoS_2_; Fermi edge of (b) CdS, (d) CdS-MXe, (f) CdS-MoS_2_, (h) CdS-MXe/MoS_2_ and (j) MoS_2_.

**References**

1. B. Cao, S. Wan, Y. Wang, H. Guo, M. Ou, [Q. Zhong,](https://www.sciencedirect.com/author/9745134300/qin-zhong) Highly-efficient visible-light-driven photocatalytic H_2_ evolution integrated with microplastic degradation over MXene/Zn_x_Cd_1-x_S photocatalyst, [***Journal of Colloid and Interface Science***](https://www.sciencedirect.com/journal/journal-of-colloid-and-interface-science), 605 (**2022**), 311-319, <https://doi.org/10.1016/j.jcis.2021.07.113>.
2. Y. Wan, S. Du, C. Lu, K. Ren, B. Shi, S. Liu, C. Li, W. Dou, P. Fang, N. Ye, Metallic CuS decorated CdS nanowires for efficient photocatalytic H_2_ evolution under visible-light irradiation, [***Journal of Alloys and Compounds***](https://www.sciencedirect.com/journal/journal-of-alloys-and-compounds), 871 (**2021**), 159461. <https://doi.org/10.1016/j.jallcom.2021.159461>.
3. U. Sahoo, S. Pattnayak, S. Choudhury, P. Aparajita, D. K. Pradhan, G. Hota, Facile synthesis of defect induced CeO_2_/MIL-53(Fe) nanocatalyst: Strategically switching the charge transfer dynamics for remarkable enhancement of photocatalytic Bisphenol A degradation and H_2_ evolution, [***Applied Catalysis B: Environmental***](https://www.sciencedirect.com/journal/applied-catalysis-b-environment-and-energy), 343 (**2024**), 123524, <https://doi.org/10.1016/j.apcatb.2023.123524>.
4. D. Ren, Z. Liang, [Y. H. Ng](https://www.sciencedirect.com/author/57205343588/yun-hau-ng), P. Zhang, [Q. Xiang](https://www.sciencedirect.com/author/26659282000/quanjun-xiang), [X. Li,](https://www.sciencedirect.com/author/57225159668/xin-li) Strongly coupled 2D-2D nanojunctions between P-doped Ni_2_S (Ni_2_SP) cocatalysts and CdS nanosheets for efficient photocatalytic H_2_ evolution, [***Chemical Engineering Journal***](https://www.sciencedirect.com/journal/chemical-engineering-journal), 390 (**2020**), 124496. <https://doi.org/10.1016/j.cej.2020.124496>.
5. L. Tian, S. Min, F. Wang, Integrating noble-metal-free metallic vanadium carbide cocatalyst with CdS for efficient visible-light-driven photocatalytic H_2_ evolution, [***Applied Catalysis B: Environmental***](https://www.sciencedirect.com/journal/applied-catalysis-b-environment-and-energy), 259 (**2019**), 118029, <https://doi.org/10.1016/j.apcatb.2019.118029>.
6. J. X. Yang, W. B. Yu, C. F. Li, W. D. Dong, L. Q. Jiang, N. Zhou, Z. P. Zhuang, J. Liu, Z. Y. Hu, H. Zhao, Y. Li, L. Chen, J. Hu, [B. L. Su,](https://www.sciencedirect.com/author/57092452600/bao-lian-su) PtO nanodots promoting Ti_3_C_2_ MXene in-situ converted Ti_3_C_2_/TiO_2_ composites for photocatalytic hydrogen production, ***Chemical Engineering Journal***, 420 (**2021**), 129695. <https://doi.org/10.1016/j.cej.2021.129695>.
7. R. Xiao, C. Zhao, Z. Zou, Z. Chen, L. Tian, H. Xu, H. Tang, Q. Liu, Z. Lin, X. Yang, In situ fabrication of 1D CdS nanorod/2D Ti_3_C_2_ MXene nanosheet Schottky heterojunction toward enhanced photocatalytic hydrogen evolution, [***Applied Catalysis B: Environmental***](https://www.sciencedirect.com/journal/applied-catalysis-b-environment-and-energy), 268 (**2020**), 118382, <https://doi.org/10.1016/j.apcatb.2019.118382>.
8. Z. Wang, Z. Qi, X. Fan, D. Y. C. Leung, J. Long, [Z. Zhang](https://www.sciencedirect.com/author/23571300200/zizhong-zhang), T. Miao, S. Meng, S. Chen, [X. Fu,](https://www.sciencedirect.com/author/55586733800/xianliang-fu) Intimately Contacted Ni_2_P on CdS Nanorods for Highly Efficient Photocatalytic H_2_ Evolution: New Phosphidation Route and the Interfacial Separation Mechanism of Charge Carriers, ***Applied Catalysis B: Environmental***, 281 (**2021**), 119443, <https://doi.org/10.1016/j.apcatb.2020.119443>.
9. S. Jin, H. Jing, L. Wang, Q. Hu, A. Zhou, Construction and performance of CdS/MoO_2_@Mo_2_C-MXene photocatalyst for H_2_ production. ***Journal of Advanced Ceramics***, 11 (**2022**), 1431–1444 (2022). <https://doi.org/10.1007/s40145-022-0621-3>.
10. Y. Wang, C. Liu, C. Kong, F. Zhang, Defect MoS_2_ and Ti_3_C_2_ nanosheets co-assisted CdS to enhance visible-light driven photocatalytic hydrogen production, [***Colloids and Surfaces A: Physicochemical and Engineering Aspects***](https://www.sciencedirect.com/journal/colloids-and-surfaces-a-physicochemical-and-engineering-aspects), 652 (**2022**), 129746, <https://doi.org/10.1016/j.colsurfa.2022.129746>.
11. [W. Zhao](https://onlinelibrary.wiley.com/authored-by/Zhao/Wenxue), [A. Yan](https://onlinelibrary.wiley.com/authored-by/Yan/Aihua), [Z. Su](https://onlinelibrary.wiley.com/authored-by/Su/Zigao), [F. Huang](https://onlinelibrary.wiley.com/authored-by/Huang/Fei), [Q. Wang](https://onlinelibrary.wiley.com/authored-by/Wang/Quande), [S. Li](https://onlinelibrary.wiley.com/authored-by/Li/Shihang), [S. Lu](https://onlinelibrary.wiley.com/authored-by/Lu/Shijian), [C. Wang](https://onlinelibrary.wiley.com/authored-by/Wang/Chuanjian), [T. Zhang](https://onlinelibrary.wiley.com/authored-by/Zhang/Tongyang), [J. Zhang](https://onlinelibrary.wiley.com/authored-by/Zhang/Jixu), [Y. Gao](https://onlinelibrary.wiley.com/authored-by/Gao/Ye), [H. Yuan](https://onlinelibrary.wiley.com/authored-by/Yuan/Huaqi), Multiobjective-Optimization MoS_2_/Cd-ZnIn_2_S_4_/CdS Composites Prepared by In Situ Structure-Tailored Technique for High-Efficiency Hydrogen Generation, ***Small Structures***, 5 (**2024**), 2300569.<https://doi.org/10.1002/sstr.202300569>.
12. J. Xu, X. Yan, Y. Qi, Y. Fu, C. Wang, L. Wang, Novel phosphidated MoS_2_ nanosheets modified CdS semiconductor for an efficient photocatalytic H_2_ evolution, [***Chemical Engineering Journal***](https://www.sciencedirect.com/journal/chemical-engineering-journal), 375 (**2019**), 122053, <https://doi.org/10.1016/j.cej.2019.122053>.
13. X. Liu, B. Wang, Q. Heng, W. Chen, X. Li, [L. Mao](https://www.sciencedirect.com/author/7202629642/liqun-mao), [W. Shangguan,](https://www.sciencedirect.com/author/7004379127/wenfeng-shangguan) Promoted charge separation on 3D interconnected Ti_3_C_2_/MoS_2_/CdS composite for enhanced photocatalytic H_2_ production, [***International Journal of Hydrogen Energy***](https://www.sciencedirect.com/journal/international-journal-of-hydrogen-energy), 47 (**2022**), 8284-8293, <https://doi.org/10.1016/j.ijhydene.2021.12.180>.
